# Pharmacological Monotherapy for Depressive Disorders: Current and Future—A Narrative Review

**DOI:** 10.3390/medicina61040558

**Published:** 2025-03-21

**Authors:** Keming Gao, Evrim Bayrak Oruc, Buket Koparal

**Affiliations:** 1 Department of Mind and Body Medicine, Sichuan Lansheng Brian Hospital, Chengdu 610036, China; 2 Department of Psychiatry, University Hospitals Cleveland Medical Center, Cleveland, OH 44106, USA; buketsevinc@gazi.edu.tr; 3 Case Western Reserve University School of Medicine, Cleveland, OH 44106, USA; 4 Independent Researcher, Cleveland, OH 44113, USA; evrimbayrakmd@gmail.com; 5 Department of Psychiatry, Gazi University School of Medicine, Ankara 06500, Turkey

**Keywords:** depressive disorders, monotherapy, antidepressants, N-methyl-D-aspartate receptor antagonists, neurosteroid antidepressants, psychedelics, opioid receptor antagonists, orexin receptors antagonists, anti-inflammatory agents, biomarker antidepressant therapy

## Abstract

*Objective*: To narratively review currently available antidepressants and future potential antidepressants as monotherapy for the treatment of depressive disorders. *Methods*: Selective serotonin reuptake inhibitors (SSRIs), serotonin norepinephrine reuptake inhibitors (SNRIs), dopamine reuptake inhibitor (bupropion), tricyclic antidepressants (TCAs), and monoamine oxidase inhibitors (MAOIs) were reviewed according to the results from Sequenced Treatment Alternatives to Relieve Depression (STAR*D) Study and systematic reviews. For the rest of the antidepressants, a PubMed/Medline search was conducted with priority for systematic reviews. For drugs in development for depressive disorders, PubMed, Google, and Clinicaltrials.gov databases were used. *Results*: The STAR*D Study demonstrated that sertraline, venlafaxine, and bupropion monotherapy had similar efficacy in patients with major depressive disorder (MDD) who failed citalopram. A network meta-analyses of randomized, placebo-controlled trials found that SSRIs, SNRIs, bupropion, TCAs, mirtazapine, and agomelatine had similar relative efficacy compared to placebo, but had different acceptability. Gepirone had more failed/negative studies and smaller effect size relative to placebo compared to other antidepressants. The combination of dextromethorphan and bupropion, ketamine infusion, and intranasal esketamine had faster onset of action but similar effect size compared to monoamine-based antidepressants as monotherapy. Brexanolone and zuranolone are effective in postpartum depression (PPD), but the effect size of zuranolone in MDD as monotherapy or adjunctive therapy was very small. Psychedelics, glutamate receptor-related agents, kappa opioid receptor antagonists, orexin receptor antagonists, new anti-inflammatory agents, and biomarker-based antidepressant therapy have been under investigation for depressive disorders. Psychedelics showed faster onset of action, large effect size, and long durability. *Conclusions*: Monoamine-based antidepressants likely continue to be the mainstream antidepressants for depressive disorder. NMDA receptor antagonists and neurosteroid antidepressants will play a bigger role with the improvement of accessibility. Psychedelics may become a game changer if phase III studies validate their efficacy and safety in depressive disorders.

## 1. Introduction

The global impact of depressive disorders is tremendous. In 2019, depressive disorders were ranked #13th of all ages in disability adjusted life years (DALYs) and #2nd in years lived with disability (YLDs) among 369 diseases [1,2]. Clinical studies have shown that correct diagnosis and adequate treatment of depressive disorders can improve functioning and prevent disability [3,4]. Currently available treatment options for depressive disorders include pharmacological agents, somatic interventions (electroconvulsive therapy, repetitive transcranial magnetic stimulation, vagus nerve stimulation, and deep brain stimulation), and psychotherapies [5]. Traditional pharmacological monotherapy agents include selective serotonin reuptake inhibitors (SSRIs), serotonin–norepinephrine reuptake inhibitors (SNRIs), dopamine reuptake inhibitors, tricyclic antidepressants (TCAs), monoamine oxidase inhibitors (MAOIs), and atypical antidepressants.

Although these medications have played a critical role in managing depressive disorders for decades, their slow onset of action and limited efficacy have propelled researchers to search for more efficacious and faster-acting antidepressants. The approval of dextromethorphan and bupropion combination (AXS-05) for non-treatment-resistant major depressive disorder (NTRMDD), intranasal esketamine for treatment-resistant depression (TRD), and brexanolone/zuranolone for postpartum depression (PPD) has reflected the success of these efforts.

Dextromethorphan, ketamine, and esketamine are noncompetitive N-methyl-D-aspartate (NMDA) receptor antagonists and brexanolone/zuranolone are neurosteroids. The antidepressant effect of AXS-05 and ketamine/esketamine is involved with the glutamatergic system through NMDA receptor antagonism. Brexanolone/zuranolone exert their effect by modulating gamma-aminobutyric acid (GABA) receptors. The AXS-05, ketamine/esketamine, and zuranolone show a faster onset of action compared to commonly used monoamine-based antidepressants.

However, the efficacy of AXS-05 and ketamine/esketamine was similar to traditional antidepressants [5]. The effect size of zuranolone monotherapy in major depressive disorder (MDD) was so small that the United States Food and Drug Administration (US FDA) denied its application for MDD. Since antidepressant monotherapy is a standard practice for patients experiencing the first or recurrent depressive episodes of MDD when they initially encounter clinicians, the aim of this narrative review is to focus on currently available antidepressant monotherapies and explore new drugs under investigation as monotherapy for MDD in the future.

## 2. Methods

Except for gepirone (Exxua; Fabre-Kramer Pharmaceuticals Inc., Malvern, PA, USA) monoamine-based antidepressants and medicinal and nutritional supplemental agents are well known and have been used for decades; the review of their mechanisms of action, efficacy, and safety was primarily based on previous publications from the Sequenced Treatment Alternatives to Relieve Depression (STAR*D) Study [6,7,8,9], a key network meta-analysis [10], and the author’s previous publications [5,11,12].

For STAR*D studies, a PubMed search was conducted with the keyword of “Sequenced Treatment Alternatives to Relieve Depression” and a further search was conducted with the names of key investigators of the STAR*D. Only original articles related to the efficacy and safety of antidepressants in adults were used for this current review. Other publications including subgroup analysis, post hoc analysis, response prediction analysis, genetic/genomic analysis, gender/race analysis, and results in adolescents were excluded.

For systematic review and meta-analysis, PubMed was searched with the keywords of “antidepressant, MDD, and meta-analysis” from 1 January 2018 to 10 March 2025. Publications included antidepressant monotherapies from different classes of antidepressants in MDD, and only publications related to the efficacy and safety of multiple antidepressants were included in this review.

For gepirone, medicinal and nutritional supplemental agents, agomelatine, NMDA receptor antagonists, neurosteroids, opioid receptor antagonists, and anti-inflammatory drugs, a PubMed search was conducted for each antidepressant with keywords of the name of a medication, MDD, or depression. All searches were conducted up to 10 March 2025. Publications related to the efficacy and safety of an antidepressant were further examined for possible inclusion in this review. The most recent systematic reviews, meta-analyses, and phase III studies of an individual medication were prioritized.

For agents currently under investigation for depressive disorders, including psychedelics, opioid receptor antagonists, orexin receptor antagonists, anti-inflammatory agents, and biomarker-based treatments, a search in the PubMed dataset was first conducted. This would provide the public available data for a medication that are peer-reviewed.

Since the new results of a studied medication, or the initiation of a new drug or a study are commonly announced through press releases, a Google search was conducted for each medication mentioned in original articles or reviews from PubMed searches. This approach provided insight into the progress of each medication and some primary findings.

Although clinical trials are required to be registered on an official website, posting the results of a study is a low priority for any sponsors of the study. Therefore, a search in the Clinicaltrials.gov database for a specific drug was conducted after the Google search, which would provide information on registered trials of a drug and the progress of each trial.

For searching psychedelics, keywords of “MDD” or “depression”, “psychedelic”, “ketamine” or “esketamine”, “psilocybin”, “methylenedioxymethamphetamine (MDMA)”, “Lysergic Acid Diethylamide (LSD)”, “5-methoxy-N, N-dimethyltryptamine (5-MeO-DMT)”, or “N, N-Dimethyltryptamine (DMT)”, and “clinical trials” were used. For searching opioid receptor antagonists, keywords of “MDD” or “depression”, “opioid receptor antagonist”, “navacaprant”, or “aticaprant”, and “clinical trials” were used. For orexin receptors antagonists, “seltorexant”, “filorexant”, “lemborexant”, “suvorexant”, “daridorexant”, “almorexant”, “TS-142” were searched in a similar way. For anti-inflammatory agents, “anti-inflammation”, “MDD” or “depression”, and “clinical trials” were used for the initial search, which was followed by a search for individual anti-inflammatory agents.

## 3. Results

### 3.1. Current Available Antidepressants as Monotherapy

#### 3.1.1. Monoamine-Based Antidepressants

A PubMed search with the term “Sequenced Treatment Alternatives to Relieve Depression” generated 291 publications. However, none were related to the original primary outcomes. Cross-referencing with a previous publication [5], four publications related to commonly used antidepressants in the STAR*D were identified and included in this review [6,7,8,9]. A PubMed search for a systematic review and meta-analysis from 1 January 2018 to 10 March 2025 generated 493 publications. Only three network meta-analyses included more than two different classes of antidepressants were included in this review [10,13,14]. A PubMed search with gepirone and major depressive disorder found 38 publications. Three of them were included in this review [15,16,17].

##### Serotonin Reuptake Inhibitors and Related Medications

SSRIs are the most prescribed antidepressants for depressive disorders. Although SSRIs share a common mechanism of action by blocking the reuptake of serotonin through occupying pre-synaptic serotonin transporters, some SSRIs at higher doses also block the reuptake of other neurotransmitters (Table 1). Some newer antidepressants, such as vilazodone and vortioxetine, also block serotonin reuptake in addition to 5-hydroxyltryptamine (5-HT) receptor agonism or antagonism (Table 1). In terms of antidepressant efficacy, there is no significant difference among SSRIs [10,14]; however, some of them have better acceptability than others, with fluoxetine having the best acceptability [10]. The side effect profiles of SSRIs are also different [5,13]. SSRIs are the first-line antidepressants for depressive disorders. They were used in the Level 1 and Level 2 treatments in the STAR*D [8,9], and the dosage of citalopram was 60 mg per day. However, the current FDA-recommended dose limit of citalopram is 40 mg per day due to its potential for QTc prolongation. An electrocardiogram (ECG) may be indicated for patients who need more than 40 mg per day.

##### Serotonin Norepinephrine Reuptake Inhibitors

SNRIs include venlafaxine, desvenlafaxine, duloxetine, milnacipran, and levomilnacipran. However, their affinities for serotonin and norepinephrine pre-synaptic transporters are different (Table 1). To achieve an SNRI antidepressant effect, at least 150 mg or more of venlafaxine should be used per day. SNRIs are also first-line antidepressants for depressive disorders. Venlafaxine was used in the Level 2 treatment in the STAR*D, and its efficacy in patients who failed citalopram was similar to that of sertraline and bupropion [8]. In placebo-controlled trials, venlafaxine and duloxetine showed a larger difference in response rates compared to placebo than SSRIs did [10,14], which is consistent with the results of Level 2 treatments in the STAR*D. In the Level 2 treatments of the STAR*D, about 25% of patients who received venlafaxine achieved remission compared to about 18% of patients who received sertraline achieving remission [8].

##### Dopamine Reuptake Inhibitor

Bupropion is a dopamine reuptake inhibitor and blocks norepinephrine reuptake at higher doses (Table 1). In the Level 2 treatments of the STAR*D, bupropion monotherapy had a similar remission rate (21%) as venlafaxine (25%) and sertraline (18%) monotherapy in patients who failed citalopram monotherapy [8]. The odds ratio (OR) for response with venlafaxine relative to placebo was 1.78 and the OR for response with bupropion relative to placebo was 1.58 [10]. Due to the overlap of the 95% confidence intervals of the ORs of venlafaxine and bupropion relative to placebo, the difference in response rates between venlafaxine and bupropion was not significant. It is reasonable to consider bupropion monotherapy as the first medication, especially for those who are present with severe anhedonia, lack of energy, poor concentration, and hypersomnia without much anxiety. Since bupropion does not have sexual side effects, for patients who have experienced sexual side effects from SSRIs and SNRIs in previous episodes, bupropion can be initiated as the first medication for a current episode as well. Bupropion is the only antidepressant that has a significantly lower risk of somnolence relative to placebo [13]. Therefore, patients with daytime somnolence/fatigue are likely to have more benefit from bupropion than from other antidepressants.

##### Norepinephrine Reuptake Inhibitor

Reboxetine is a selective norepinephrine reuptake inhibitor (Table 1) [18]. Reboxetine is mainly available in Europe, South American countries, and China [19]. However, studies conducted in the US and Canada resulted in a US FDA non-approval decision. In both network meta-analyses [10,14], reboxetine was the least effective antidepressant among 21 antidepressants. However, it has a linear increase in effect size as its doses increase [14].

##### Serotonin, Norepinephrine, and Dopamine Reuptake Inhibitors

Toludesvenlafaxine is a prodrug to desvenfafaxine and inhibits serotonin, norepinephrine, and dopamine reuptake through blocking SERT, NET, and DAT, respectively (Table 1) [20]. Its antidepressant efficacy has been demonstrated with one phase II and one phase III randomized, double-blind, placebo-controlled trial [21,22]. Currently, it is only available in China. In a recent systematic review and network meta-analysis of 22 antidepressants [14], toludesvenlafaxine had the largest OR of 4.52 relative to placebo for response. Among the rest of the antidepressants, only amitriptyline had an OR of 2.27 relative to placebo for response, and the rest had ORs of less than 2.0 for response. The larger effect size of toludesvenlafaxine relative to placebo, compared to other antidepressants, might be due to the fact that only a few studies of toludesvenlafaxine were included in the meta-analysis. More studies are needed to validate the efficacy of toludesvenlafaxine in depressive disorders.

##### Atypical Antidepressants

Although there is no standard definition for atypical antidepressants, the mechanisms of action of mirtazapine, trazodone, and nefazodone are quite different from other antidepressants (Table 1). Among these medications, trazodone is commonly used for sleep and mirtazapine is used for depression as a second-line medication similar to TCAs. However, patients treated with mirtazapine had the second highest chance for response relative to placebo among all 21 antidepressants, and the acceptability was comparable to that of placebo [10]. Mirtazapine as a second-line medication is mainly due to its side effect profiles, including higher rates of drowsiness/sedation, increased appetite/weight gain, xerostomia, increased serum cholesterol, hypertriglyceridemia, and constipation compared to SSRIs, SNRIs, and dopamine reuptake inhibitors. In addition, mirtazapine also has rare but severe side effects including thrombocytopenia, bone marrow suppression and neutropenia, and acute pancreatitis.

In the STAR*D, mirtazapine was a Level 3 treatment as nortriptyline monotherapy and augmentation therapy with lithium and T3 (triiodothyronine). There were no significant differences among these treatments, although mirtazapine had the lowest remission rate of 12% [6]. The remission rate of nortriptyline was 20%. It is worth noting that the patients in Level 3 treatments were those who had failed citalopram and sertraline, venlafaxine, bupropion, citalopram plus bupropion, or citalopram plus buspirone.

##### Tricyclic Antidepressants

TCAs include tertiary amines (amitriptyline, clomipramine, doxepin, imipramine, and trimipramine), secondary amines (desipramine, nortriptyline, and protriptyline), and tetracyclics (amoxapine and maprotiline). Their affinities for serotonin transporters, norepinephrine transporters, and 5-TH_2C_ receptors are different (Table 1). Currently, TCAs are considered as the second-line antidepressants and nortriptyline was used as a Level 3 treatment in the STAR*D [6]. In the placebo-controlled studies, amitriptyline had the highest chance of response relative to placebo among all 21 antidepressants [10]. Clomipramine was also significantly more effective than placebo and as effective as most other antidepressants. Amitriptyline and clomipramine had a linear increase in effect size as their doses increased [14]. However, clomipramine had the worst acceptability [10].

The effective blood levels of amitriptyline, desipramine, doxepin, imipramine, and nortriptyline are established; however, nortriptyline’s level has a bell shape with a narrow window of efficacy. For patients who are indicated for a tricyclic for MDD, monitoring the blood level may be necessary. Due to their atrioventricular (AV) block and quinidine-like antiarrhythmic effects, all patients considering a TCA should be screened for preexisting cardiac conditions, including prolonged QTc intervals, heart disease, and a family history of arrhythmias. A baseline and periodical ECG are essential, especially for those who have cardiac diseases and other cardiac risk factors. More importantly, intentional or accidental overdose of a TCA can be fatal. Careful assessment of suicidal risk and close monitoring of the progress of patients are critical to minimize the risk of intentional or accidental death.

TCAs may be more effective than SSRIs in MDD with melancholic features. A TCA may be considered for this subgroup of patients when an SSRI fails. However, TCAs affect cholinergic and histaminergic systems in addition to serotonin and norepinephrine. Therefore, TCAs have many more side effects than SSRIs, SNRIs, and bupropion. In addition, suddenly stopping TCAs may cause withdrawal symptoms (cholinergic rebound) including excessive salivation, diarrhea, headache, and vivid dreams.

##### MAOI Antidepressants

In the STAR*D, only about 7% of patients who failed nortriptyline, mirtazapine, or augmentation with lithium or T3 (Level 3 treatments) achieved remission with tranylcypromine, a non-selective and irreversible MAOI [7]. In the network meta-analysis of 21 antidepressants, no study of MAOIs in MDD was included [10]. The lack of a randomized, placebo-controlled or double-blind head-to-head comparison study of MAOIs in MDD might be due to their potential life-threatening side effect such as hypertensive crisis. In addition to interactions with fermented food and drinks, MAOIs can also interact with other antidepressants, which leads to potential severe consequences. When switching from an MAOI to another antidepressant, patients should wait for at least 14 days before initiating the new treatment to prevent any drug-to-drug interactions. Similarly, when switching from an SSRI, SNRI, or TCA to an MAOI, patients should wait for at least two weeks before initiating the new treatment. However, for those who take fluoxetine, they should wait for five weeks before starting an MAOI.

##### Gepirone

Gepirone is a 5-HT_1A_ partial agonist and 5-HT_2A_ antagonist and it is in the same family as buspirone but has three times higher affinity for the 5-HT_1A_ receptor [17]. Gepirone extended release (ER) was approved by the US FDA for the treatment of NTRMDD in 2023. The approval process took more than 20 years and involved multiple submissions because gepirone in NTRMDD trials had much more negative/failed studies (87%) than positive studies. In contrast, most other US FDA-approved antidepressants have about 49% negative/failed trials [23]. There were two positive studies supporting the efficacy of gepirone-ER for the treatment of NTRMDD, but the US FDA rejected the application three times due to less robust results [24]. After a series of important debates and an internal review, the US FDA finally approved gepirone-ER monotherapy for NTRMDD [15,16].

However, even in the two positive studies, the difference between gepirone and placebo in reducing the Hamilton Depression Rating Scale-17 item (HAMD-17) total score was about 2.5 points, which is smaller than the clinically meaningful difference of 3–5 points that was observed for other FDA-approved antidepressants [25]. In contrast, the number needed to treat (NNT) of 6–8 for response and 7 for remission from gepirone relative to placebo was comparable to that of the lower-performing antidepressants approved by the US FDA [23]. It remains unclear if the relative efficacy of gepirone will change if it is included in a network meta-analysis like agometaline [10,14].

Unlike other antidepressants, gepirone carries a risk of QTc prolongation with an FDA warning and recommendation of ECG monitoring during the treatment. On the other hand, gepirone has minimal sexual side effects and does not cause weight gain, so it may offer relief and improve sexual function in patients with depression like bupropion and vilazodone [23]. While gepirone represents a novel addition to monoamine-based antidepressants, its cardiac risks, limited efficacy, and FDA warnings make it unlikely to become a first-line treatment for depressive disorders.

#### 3.1.2. Melatonergic Antidepressant

A PubMed search with keywords of “agomelatine” and “depression” generated 633 publications including eleven systematic reviews and meta-analyses and three phase III studies regarding the efficacy, safety, and acceptability of agomelatine in MDD. Three meta-analyses [26,27,28], two network analyses [10,14], and a review [29] were included in this review.

##### Agomelatine

Agomelatine is an antidepressant that targets the melatonergic system and 5-HT_2C_ receptors. Its antidepressant effect is believed to work through the agonism of melatonergic receptors and the antagonism of 5-HT_2C_ receptors (Table 1). Although agomelatine was approved for the treatment of depression in Europe (2009), Australia (2010), and some other countries, it did not receive approval for NTRMDD from the US FDA. The main issue with agomelatine was its smaller effect size compared to other commonly used antidepressants. Its application for depression was rejected twice by the European Medicines Agency [29]. In an early meta-analysis of nine randomized, double-blinded, placebo-controlled studies of agomelatine versus placebo or other antidepressants (paroxetine, fluoxetine, sertraline, venlafaxine) in 3943 patients with severe NTRMDD [26], agomelatine was superior to placebo with a standardized mean difference (SMD) of −0.26, which is smaller than the overall antidepressant effect size of −0.3 [30]. The meta-analysis also found a slight superiority of agomelatine over other antidepressants with an SMD of −0.11. In the network analysis of 21 antidepressants, agomelatine was significantly superior to placebo in response rate, as most other antidepressants, and had the best acceptability [10]. Head-to-head comparison studies also suggested that agomelatine along with amitriptyline, escitalopram, mirtazapine, paroxetine, venlafaxine, and vortioxetine was more effective than other antidepressants. Moreover, agomelatine has more benign side effects with minimal sexual side effects compared to other antidepressants, and has hypnotic effects.

#### 3.1.3. Medicinal and Nutritional Supplemental Agents

A PudMed search with keywords of “hypericum perforatum” and “depression” generated 897 publications including sixteen systematic reviews and meta-analyses and one phase III study. Two meta-analyses were included in this review [31,32]. For “S-Adenosyl-L-Methionine” and “L-methyl folate”, there were 440 publications discovered with the PubMed search, including eighteen systematic reviews and meta-analyses and two phase III trials. One review [33], three systematic reviews and meta-analyses [34,35,36], two phase III trials, and one open-naturalistic trail [37] were included in this review. For Omega-3 fatty acids, there were 1612 publications, including thirty-one meta-analyses and systemic reviews, and two of them were included in this review [38,39].

##### St. John’s Wort

St. John’s Wort (*Hypericum perforatum*) is an herb that contains many bioactive molecules. It has been used as a homeopathic remedy for different illnesses for decades [40]. Its antidepressant effect is involved with glutamate, acetylcholine, serotonin, norepinephrine, and dopamine [41]. Different studies on St. John’s Wort used different doses and formulations, which might have caused the inconsistent results of St. John’s Wort versus placebo. Meta-analyses found that St. John’s Wort was superior to placebo in mild and moderate depression, and there were no significant differences between second generation antidepressants (SGAs) and St. John’s Wort [31,32]. However, all head-to-head comparison trials of St. John’s Wort versus SGAs used low to moderate doses of SGAs. The overall risk of adverse events was higher among patients receiving SGAs than those receiving St. John’s Wort, but the risk of serious adverse events did not differ significantly.

##### S-Adenosyl-L-Methionine and L-Methyl Folate

S-Adenosyl-L-Methionine (SAMe) and folate are naturally occurring compounds that are present in nearly all tissues in the body. Their role in depressive disorders is based on observations that metabolites from one-carbon metabolism play an important role in the pathogenesis and treatment of depression [33,34,42]. Both SAMe and folate are key components of the one-carbon metabolic cycle (Table 1). Currently, L-methyl folate and SAMe monotherapy or adjunctive therapy to an antidepressant have been studied in the treatment of NTRMDD and TRD. L-methyl folate is approved by the US FDA as a medicinal supplement for augmenting antidepressants in the treatment of TRD.

A recent systematic review of eight trials and 1011 subjects (three of the five were SAMe versus placebo) found that SAMe was significantly better than placebo in reducing depressive symptoms [35]. However, a Cochrane Database Systematic Review in 2016 of eight trials of SAMe versus placebo, imipramine, desipramine, or escitalopram (*n* = 934) did not find strong evidence that SAMe was superior to placebo in reducing depressive symptoms [36]. Both reviews did not find significant differences between SAMe and other antidepressants (imipramine or escitalopram). Due to the low quality of published studies so far, large randomized, double-blind, placebo or active controlled studies are necessary to confirm the effectiveness of SAMe in the treatment of NTRMDD as a monotherapy.

L-methyl folate is the only form of folate that crosses the blood–brain barrier. Its efficacy and safety in TRD were assessed in two multicenter randomized, double-blind, sequential parallel comparison trials in patients who failed one or two SSRIs. Patients received L-methyl folate 7.5 mg/day, L-methyl folate 15 mg/day, and placebo along with their ongoing antidepressant for 30 days [33]. Patients who received L-methyl folate 15 mg/day had a significant reduction in depression symptoms over those who received the placebo. The response rate in the L-methyl folate 15 mg group was also significantly higher than in the placebo group, 32.5% versus 14.6%, with an NNT for response of 6 favoring L-methyl folate 15 mg/day over the placebo. A recent meta-analysis of six randomized, placebo-controlled trials with a total number of 584 patients found that adjunctive therapy of L-methyl folate/folic acid to SSRIs/SNRIs had significantly higher rates of response and remission compared to placebo [34]. In a real-world patient study (*n* = 554), adding L-methyl folate 7.5 mg or 15 mg to the existing antidepressant treatment, 67.9% of patients achieved response, defined as a 50% reduction in baseline Patient Health Questionnaire-9 (PHQ-9) score and 253 patients (45.7%) achieved remission, defined as a PHQ-9 score < 5 after an average of 95 days of therapy [37].

##### Omega-3 Fatty Acids

The mechanism of Omega-3 fatty acids (Omega-3) in depression remains unclear. The potential mechanisms are involved with its anti-neuroinflammatory effect, regulation of the hypothalamus–pituitary–adrenal (HPA) axis, anti-oxidative stress, anti-neurodegeneration, neuroplasticity, and modulation of neurotransmitter systems [43]. An early meta-analysis of 2160 patients found that compared with placebo, 100% eicosapentaenoic acid (EPA) and ≥60% EPA-major formulations with an EPA dosage ≤ 1 g/day were significantly superior to placebo in reducing depressive symptoms. However, pure docosahexaenoic acid (DHA) and DHA-major formulations did not significantly differ from placebo [39]. A Cochrane Database Systematic Review of 34 studies with 1924 patients found that Omega-3 supplementation results in a small to modest benefit in improving depressive symptoms with an SMD of −0.40. Overall, the quality of the evidence for all outcomes was rated low to very low [38].

#### 3.1.4. NMDA Receptor-Related Medications

A PubMed search with keywords of “dextromethorphan and bupropion combination” and “depression” generated thirty-seven publications, including one network meta-analysis and two systematic reviews, one phase II study, and one phase III trials. Both phase II and phase III trials were included in this review [44,45]. For intranasal esketamine, a PubMed search with terms of “esketamine” and “depression” generated 1075 publications, including six systematic reviews and meta-analyses for both ketamine infusion and intranasal esketamine, seven meta-analyses for intranasal esketamine alone, and ten phase III trials of intranasal esketamine in MDD. In this review, four systematic review and meta-analyses of intranasal esketamine [46,47,48,49], and six phase III trials [50,51,52,53,54,55,56] were included. For ketamine infusion, there were 998 publications generated through a PubMed search with keywords “ketamine infusion” and “depression”. Among these, nine systematic reviews and meta-analyses focused on ketamine infusion alone, while six included both ketamine infusion and intranasal esketamine. We included three studies with different study designs and different patient populations because there was no standardized ketamine infusion protocol [57,58,59].

##### Dextromethorphan and Bupropion Combination (AXS-05)

AXS-05 (Auvelity, Axsome Therapeutics, New York City, NY, USA) was approved by the US FDA for the treatment of NTRMDD in adults in August 2022. Dextromethorphan is an uncompetitive NMDA receptor antagonist like ketamine that increases glutamate release as well as an agonist to sigma-1 receptor that increases serotonin levels (Table 1). The approval was based on one phase II and one phase III randomized, double-blind, placebo-controlled trial [44,45]. The studied population was patients with a DSM-5 diagnosis of MDD for at least four weeks and Montgomery–Asberg Depression Rating Scale (MADRS) scores ≥ 25 points. Eligible patients were randomized to receive AXS-05 monotherapy or placebo for six weeks. In a phase III study [45], the significant superiority of AXS-05 over placebo occurred as early as week 1 with a difference of 2.23 points on MADRS total scores, week 2 with a difference of 4.22 points, and week 6 with a difference of 3.9 points favoring AXS-05. The remission (MADRS total score of ≤10) rate was significantly higher in the AXS-05 group, with a difference of 9.4% at week 2 and a difference of 22.2% at week 5. For treatment response (≥50% reduction in MADRS total score), patients who received AXS-05 had significantly higher response rates than the placebo group at all time points, with a difference of 20% at week 6. Adverse events occurring in ≥5% of patients in the AXS-05 group were dizziness, nausea, headache, diarrhea, somnolence, and dry mouth. AXS-05 was not associated with psychotomimetic effects, weight gain, or sexual dysfunction. Since dextromethorphan increases serotonin levels, AXS-05 should be used with caution when monoamine-based antidepressant(s) and other serotonin-increasing agents are used.

##### Intranasal Esketamine

Esketamine (S-ketamine) is more potent than the racemic ketamine that is a mixture of R-ketamine and S-ketamine. The bioavailability of intranasal esketamine (SPRAVATPO, Janssen Pharmaceuticals, Titusville, NJ, USA) is approximately 50%. Intranasal esketamine plus an antidepressant was approved by the US FDA on 5 March 2019 for the treatment of TRD, and intranasal esketamine monotherapy for TRD was approved on 21 January 2025. The US FDA also approved intranasal esketamine to treat adult patients with MDD and acute suicidal ideation (SI) or behavior on 3 August 2020.

The approval of intranasal esketamine as an adjunctive therapy for TRD was mainly based on three acute phase III studies [50,51,52], and one long-term phase III study [53]. Among the three acute phase III studies, only one of them demonstrated that intranasal esketamine plus an antidepressant was superior to placebo plus an antidepressant in reducing depressive symptoms [51]. The other two studies did not show the superiority of intranasal esketamine over placebo in the primary outcome, but some secondary outcomes showed significant differences in favor of esketamine. One of the long-term studies also favored esketamine over placebo. The effect size for the change in MADRS score from baseline to day 28 was 0.30 (small), even in the positive study.

The approval of intranasal esketamine as a monotherapy for TRD was mainly based on a phase IV double-blind, placebo-controlled, multiple-center study in patients with MDD who had failed ≥ 2 oral antidepressants (≤25% improvement) based on treatment history for the current episode. Intranasal esketamine, both 56 mg and 84 mg, was superior to placebo in reducing depressive symptoms in 4 weeks and the onset of action occurred as early as 24 h (NCT04599855) [54]. In a post hoc analysis, the remission rates (MADRS score ≤ 12) were 22.5% for intranasal esketamine and 7.6% for placebo. The approval of esketamine as a monotherapy for TRD provides an option for patients who cannot tolerate an antidepressant.

The approval for adults with MDD and acute SI and behavior was mainly based on two phase III studies [55,56]. Both studies demonstrated that intranasal esketamine plus standard of care antidepressant therapy (SAT) was superior to placebo plus SAT in reducing depressive symptoms’ severity. The superiority started as early as four hours after receiving the first dose of esketamine and was sustained until the end of the 25-day study period. However, the change in suicidality severity was not significantly different between the two groups, although the esketamine plus SAT group had more reductions in suicidality than the placebo plus SAT group.

An earlier meta-analysis of four studies with three TRD studies found that a significant difference between esketamine and placebo in response and remission started as early as 2 h after the first dose, peaked at 24 h, and persisted until the end of the study [46]. The NNT of response with three TRD studies was 7 while the NNT of remission (MADRS ≤ 12 or 10) was 9. A more recent meta-analysis of nine studies of esketamine in patients with TRD or MDD with SI and intent found that the difference in remission rate between esketamine and placebo was significantly different, with a risk ratio (RR) of 1.35 with 84 mg and an RR of 1.66 with flexible doses [47]. However, there was no significant difference in remission rates between esketamine 28 or 56 mg and placebo. Common side effects of esketamine included dissociation, vertigo, nausea, dizziness, dysgeusia, headache, somnolence, hypoesthesia, paresthesia, and oral hypoesthesia [50,51,52].

Intranasal esketamine therapy starts twice a week for four weeks, with a first session of 56 mg and seven sessions of 84 mg. Patients who have at least 50% improvement after the first eight sessions continue treatment with the same dose as the last session and at a weekly interval for 4 weeks and biweekly twice. From then on, the treatment interval for future sessions will be determined by the mood stability of patients.

During each session, patients are closely monitored for 2 h after dosing. After each treatment, patients should not engage in potentially hazardous activities such as driving a motor vehicle and operating machinery until the next day after a restful sleep.

##### Ketamine Infusion

Ketamine infusion commonly uses racemic ketamine that is a mixture of R-ketamine and S-ketamine. Ketamine is a noncompetitive NMDA receptor antagonist. Its antidepressant effect is believed to occur through blocking NMDA receptors on pre-synaptic GABAergic interneurons which regulate the release of glutamate from pre-synaptic glutamatergic neurons. Increased glutamate in the synaptic cleft acts on post-synaptic neurons, leading to an increase in the production of brain-derived neurotrophic factor (BDNF), neuroplasticity, and synaptogenesis through activating NMDA receptors and α-amino-3-hydroxy-5-methyl-4-isoxazolepropionic acid (AMPA) receptors [60]. In addition, ketamine and other noncompetitive NMDA receptor antagonists may restore synaptic homeostasis through blocking pathologically hyperactive glutamate N2D subtypes of NMDA receptors in patients with MDD [61] (Table 1).

Most studies on ketamine infusion in depression have been conducted in patients with TRD or treatment-resistant bipolar depression using a single infusion. A rapid onset of antidepressant effects (within four hours) with a large effect size was observed in normal saline-controlled or midazolam-controlled studies [57,58,59,62]. However, there is no standard protocol for ketamine infusion, although most academic centers have used 0.5 mg/kg to 0.75 mg/kg in 40–60 min two to three times a week for treatment-resistant bipolar or unipolar depression [60,63]. Some clinicians may use doses up to 1.5 mg/kg or higher in 40–60 min for treatments based on treatment response and side effects. There is also no consensus on the optimal frequency and duration following an acute series of ketamine infusions. It remains unclear whether ketamine infusion for depression can be used as a monotherapy for NTRMDD.

#### 3.1.5. Neurosteroid Antidepressants

A PubMed search with keywords of “brexanolone” and “depression” found 159 publications, including four systematic reviews and meta-analyses and two phase III trials. We used two phase III trials for this review [64,65]. For “zuranolone”, we found 106 publications, including six systematic reviews and meta-analyses and six phase III trials, with six of them being included for this review [66,67,68,69,70,71].

##### Brexanolone in PPD

Brexanolone (Zulresso, Sage Therapeutics, Cambridge, MA, USA) is an aqueous formulation of allopregnanolone, which is a major metabolite of progesterone, and it was approved by the US FDA for PPD with a Risk Evaluation and Mitigation Strategy (REMS) in 2019. Its mechanism of action is believed to change neuronal excitability through modulating GABA receptors, especially synaptic and extra synaptic GABA_A_ receptors [72]. The approval was based on two phase III studies which enrolled women aged 18 to 45 years with PPD [64,65]. Eligible participants were randomized to receive a single 60 h continuous infusion of either brexanolone 90 μg/kg/h, brexanolone 60 μg/kg/h, or placebo [64]. The mean difference in change from baseline in HAMD-17 score between the placebo group and both brexanolone infusion groups was statistically significant. Remission rates at 60 h were significantly higher in the brexanolone 60 μg group compared to the placebo group (51.4% versus 16.3%), but not significant in the brexanolone 90 μg group compared to placebo (30.8% versus 16.3%). The response rates were significantly higher in both groups compared to the placebo group at 60 h, with 86.5% in the brexanolone 60 μg group, 74.4% in the brexanolone 90 μg group, and 55.8% in the placebo group. Brexanolone infusion was generally well tolerated; the most common treatment-emergent adverse events (AEs) in the brexanolone groups were tachycardia, headache, dizziness, and somnolence. The second study compared the efficacy of brexanolone 90 μg/kg/h and placebo and showed similar findings [65].

Under the REMS, healthcare facilities and pharmacies are required to be certified, and patients are required to enroll before receiving brexanolone infusion. The goal of these requirements is to mitigate the potential risk of serious harm resulting from excessive sedation and sudden loss of consciousness during the infusion. The concentration of brexanolone varies during a 60 h infusion: 0–4 h at 30 mcg/kg/hour, increases to 60 mcg/kg/hour during 4–24 h, increases to 90 mcg/kg/hour during 24–52 h, decreases to 60 mcg/kg/hour during 52–56 h, and decreases to 30 mcg/kg/hour during 56–60 h. Because of the REMS requirement and 60 h continuous infusion, brexanolone has very limited use in routine clinical practice.

##### Zuranolone in PPD

Zuranolone (Zurzuvae, Sage Therapeutics and Biogen, Cambridge, MA, USA) (is a synthetic, oral neuroactive steroid that acts as a positive allosteric modulator on GABA_A_ receptors, similar to brexanolone, and it was approved by the US FDA for PPD on 4 August 2023. The approval was based on the results of two phase III positive studies of zuranolone versus placebo in PDD [66,67]. In a phase III study [66], eligible patients were randomized to receive placebo (*n* = 76) or oral zuranolone 30 mg (*n* = 77) each evening for two weeks [66]. Zuranolone had a significant reduction in HAMD-17 total scores from baseline to day 15 compared to placebo, with a 4.2-point mean difference. The significant differences between zuranolone and placebo were observed from day 3 through day 45.

Response rates were significantly higher in the zuranolone group than in the placebo, with 72% vs. 48% at day 15 and 75% vs. 57% at day 45, respectively. Remission rates (HAMD-17 ≤ 7) were also significantly higher in the zuranolone group than in the placebo group, with 19% vs. 5% at day 3, and 45% vs. 23% at day 15, respectively. The race, age, stable concomitant antidepressant, body mass index (BMI), onset of PPD, and family history of PPD did not affect the efficacy of zuranolone. The second phase III study using zuranolone 50 mg/day versus placebo showed similar results [67]. Zuranolone was well tolerated. The common side effects with higher rates in the zuranolone group were somnolence, dizziness, upper respiratory tract infection, diarrhea, and sedation.

Currently, the US FDA recommends zuranolone 50 mg at evening with fat-containing food daily for 14 days. A lower dose is an option for those who cannot tolerate 50 mg/day. Due to a higher risk for somnolence from zuranolone 50 mg relative to placebo (36% vs. 6%), patients should not drive a motor vehicle or engage in other potentially hazardous activities requiring complete mental alertness such as operating machinery for at least 12 h after taking zuranolone.

##### Zuranolone for MDD

There are four phase III studies of zuranolone for the treatment of MDD [68,69,70,71]. In a phase II study [73], patients were randomized to zuranolone 30 mg (20 mg if necessary) or placebo at evening time for 2 weeks. At day 15, the zuranolone group (*n* = 45) had a significantly greater reduction in HAMD-17 scores from baseline compared to the placebo group (*n* = 44), with −17.4 points versus −10.5 points (*p* < 0.001). From day 2 to day 28, the difference between the groups in HAMD-17 was significant but not at day 35 or day 42. At day 15, the NNT for both response and remission was three. At day 3, the NNT for response and remission was five and ten, respectively. At day 28, the response and remission rates of zuranolone versus placebo were 62% vs. 46%, and 52% vs. 28%, respectively. There were also significant differences in other secondary outcome measures including changes in MADRS, Hamilton Anxiety Rating Scale (HAM-A), and Clinical Global Impression-Improvement Scale (CGI-I).

However, the rapid onset and large effect size observed from the zuranolone phase II study were not replicated in the phase III studies [73]. Overall, the efficacy of zuranolone in MDD was less robust compared to the results in PPD. Even in the positive studies, the differences between zuranolone and placebo at the end of study were very small. For this reason, the US FDA declined to approve zuranolone as a monotherapy or as an adjunctive treatment to standard antidepressants in MDD [16].

#### 3.1.6. Opioid Receptor Antagonist

A PubMed search with terms of “opioid receptor antagonist” and “depression” generated 3338 publications, including three systematic reviews and meta-analyses and three phase III trials. For this review, we used three phase III trials [74,75,76].

The endogenous opioid system plays an important role in mood regulation and reward processing. It is hypothesized that the endogenous opioid system is dysfunctional when depression occurs. The dynorphin-kappa (κ) receptor system works as an endogenous anti-reward system and κ receptor agonists have pro-depressive effects. In contrast, mu (µ) receptors and delta (ẟ) receptors activate the reward system and have antidepressant effects. Previous studies also suggested that chronic exposure to stress might activate the κ receptor system that leads to depressive-like effects. In contrast, κ receptor antagonists consistently demonstrated positive effects on reducing stress-related behaviors and showed antidepressant effects. Early small-scale studies in depression focused on mu (µ) receptor agonists or partial agonists such as morphine, methadone, codeine, and buprenorphine, and reported antidepressant effects in depressive disorders [77]. However, the abuse potential of µ receptor agonists has limited their development in the treatment of MDD.

To avoid potential abuse of buprenorphine, an opioid κ receptor antagonist and partial µ receptor agonist, the combination of buprenorphine and samidorphan (Alkermes, Inc., Waltham, MA, USA), a µ receptor antagonist (also known as ALKS5461), was explored with large randomized, double-blind, placebo-controlled trials in MDD [77]. A small study of buprenorphine/samidorphan in the treatment of TRD showed a moderate size effect on both HAMD-17 and MARDS with a 2 mg/2 mg combination, but an 8 mg/8 mg combination did not show a significant difference from the placebo [74]. However, among three phase III studies, only one showed the superiority of buprenorphine/samidorphan over placebo in reducing depressive symptoms [75,76].

#### 3.1.7. Anti-Inflammatory Drugs

A PubMed search with terms of “anti-inflammatory drugs” and “depression” found 18,567 publications, with eleven systematic reviews and meta-analyses on the efficacy and safety of anti-inflammatory drugs in depression. Celecoxib was the most studied non-steroid anti-inflammatory drug in depression, with 187 publications and four meta-analyses on efficacy and safety. Minocycline is an antibiotic for infectious and non-infectious conditions, with 287 publications and five meta-analyses in depression. We used four meta-analyses for this review [78,79,80,81].

The involvement of pro-inflammatory cytokines in MDD has been supported by an increase in cytokines in both the central nervous system and peripheral blood system in some patients with MDD [82,83]. The effort of repurposing well-known anti-inflammatory drugs for the treatment of depressive disorders has continued. A phase III study sponsored by Universiteit Antwerpen, Belgium (NCT05644301) to assess the efficacy and safety of minocycline, celecoxib, or placebo in TRD is ongoing. In the study, the patients with current MDD who fail to achieve remission with an ongoing antidepressant at an adequate dose or relapse while on an antidepressant are stratified based on the level of high sensitivity C-reactive protein (hs–CRP), <3 mg/L or >3 mg/L, and randomly assigned to minocycline, celecoxib, or placebo for 12 weeks. The study is estimated to be completed in late 2026.

A more recent meta-analysis found that anti-inflammatory agents had a significant antidepressant effect compared to placebo when used as an adjunctive therapy to antidepressants in patients with TRD and NTRMDD [78]. Generally, most studies assessed the efficacy of anti-inflammatory drugs as an adjunctive therapy to an antidepressant in patients with TRD. A small number of studies have been conducted as monotherapy in patients with depressive symptoms and inflammatory diseases. The overall impression is that anti-inflammatory drug monotherapy might be beneficial in reducing depressive symptoms in patients with inflammatory comorbidities [78,81,84].

A meta-analysis of randomized, placebo-controlled trials of celecoxib in depressive disorders found that celecoxib had a significant antidepressant effect at a dose of 400 mg/day compared to placebo as an add-on treatment in MDD with an SMD of −1.12, and as a monotherapy in depressed patients with somatic comorbidity with an SMD of −1.35 [80,85,86]. However, in a meta-analysis of minocycline in depression with eight trials of 567 participants, minocycline was not superior to placebo in reducing depressive symptoms [79]. Celecoxib at a dose of 400 mg/d used for up to 12 weeks appeared to be safe in patients with mood disorders.

### 3.2. Future Possible Monotherapy Antidepressants

#### 3.2.1. Psychedelics

Psychedelics and psychedelic-assisted psychotherapy are the most recently studied agents in the treatment of depressive disorders [87]. In addition to dissociative anesthetics, NMDA receptor antagonists (ketamine and esketamine), psilocybin, lysergic acid diethylamide (LSD), 3,4-methylenedioxymethaphetamine (MDMA), N, N-Dimethyltryptamine (DMT), and 5-methoxy-N, N-dimethyltryptamine (5-MeO-DMT) are being developed for the treatment of depressive and other psychiatric disorders [88].

##### Psilocybin

Psilocybin is a serotonergic hallucinogen that mainly exerts its effects through the partial agonism of 5-HT_2A_ receptors and binding to 5-HT_2C_, 5-HT_1A_, and 5-HT_1B_ receptors in the brain [88]. Psilocybin is the most studied psychedelic in the treatment of depressive disorders [89]. Several small-scale studies have demonstrated the efficacy of psilocybin in the treatment of depression and TRD [88,90,91]. The preliminary evidence of psilocybin in depressive disorders and the limitations of current pharmacological treatments for depressive disorder led the US FDA to grant “breakthrough therapy” status to psilocybin for the treatment of TRD in 2019 [88].

In a trial of a single, moderate dose of psilocybin (0.215 mg/kg) monotherapy versus placebo with therapeutic support in patients who had mild to severe depressive symptom severity, psilocybin yielded a significantly larger reduction in MADRS score relative to placebo with a large effect size at day 7 and a moderate effect size at day 14 after the treatment. The significant difference between psilocybin and placebo occurred 6 h after the administration. The response and remission rates were also significantly higher in the psilocybin group compared to the placebo group, with 58% versus 15% for response and 54% versus 12% for remission, respectively [92].

Currently, Compass Pathways of United Kingdom is developing psilocybin for TRD (NCT05711940 and NCT 05624268). Psilocybin is also being studied for NTRMDD (NCT 06308653) and MDD with borderline personality disorder (NCT 05399498). Some researchers are investigating the safety and efficacy of psilocybin in the treatment of patients with back pain and depression (NCT 0635541), Parkinson’s disease with depression (NCT 06455293), and mild cognitive impairment/early dementia with depression (NCT 04123314). Psilocybin-assisted cognitive behavioral therapy (CBT) for depression is also under investigation (NCT 05227612). More importantly, psilocybin as a monotherapy has shown efficacy in reducing depressive symptoms in “secondary” depression and NTRMDD [93,94,95,96,97]. Meanwhile, an open-label study of 12 patients with severe TRD (failure ≥ 5 treatments) showed that a single dose of psilocybin (COMP360) monotherapy with assisted therapy significantly reduced depressive symptom severity from baseline to week 1, week 2, week 3, week 6, week 9, and week 12 [98]. The remission and response rates at week 1 were both 75%, while at week 12, they were 25% and 58.3%, respectively. With the positive results of previous studies, psilocybin is likely to be developed for various depressive disorders as a monotherapy or adjunctive therapy.

##### Lysergic Acid Diethylamide (LSD)

Like psilocybin, the hallucinogenic effects of LSD are mainly through the partial agonism of 5-HT_2A_ receptors and binding to 5-HT_1A_, 5-HT_2C_, and 5-HT_1B_ receptors in the brain [88]. In addition, LSD also binds to dopamine D2 receptors and increases glutamate release in the frontal cortex. Currently, LSD is being developed by MindBio Therapeutics Corp (Vancouver, BC, Canada) for the treatment of MDD [99], and by Mind Medicine (MindMed) Inc. for the treatment of generalized anxiety disorder (GAD) and MDD. Previously, LSD was mainly studied in the treatment of alcohol use disorders [100,101].

In a MindMed (MindMed Therapeutics Corp., New York City, NY, USA) phase II dose-finding study of LSD (NCT05407064) with MM-120 (LSD D-Tartrate) at doses of 25, 50, 100, 200 μg, or placebo, a 100 µg dose of MM-120 yielded the largest significant difference relative to placebo, with a 7.7-point difference in HAM-A scores. MM-120 was generally well tolerated with most adverse events rated as mild to moderate, transient, and occurring on the dosing day. The findings from this phase II study made the LSD MM-120 receive “breakthrough therapy” designation from the US FDA in March 2024 [102]. Currently, a phase III trial in GAD sponsored by MindMed is ongoing (NCT06741228).

In a phase II study of LSD in MDD sponsored by University Hospital, Basel, Switzerland in collaboration with MindMed (NCT03866252), patients with MDD were randomly and blindly assigned to receive either LSD 25 µg on their first and second dosing days (low dose, placebo), LSD 100 µg at their first and second dosing days (moderate dose, active), or 200 µg on their first and second dosing days (high dose, active). The first and second dose were four weeks apart. Six weeks after the first dose administration, patients in the high dose arm (*n* = 28) had a significant reduction in Inventory of Depressive Symptomatology-Clinician (IDS-C) scores of −12.9 points compared to −3.6 points in the lower dose arm (*n* = 27). The significant benefit was maintained for up to 16 weeks after the first administration compared to placebo. The results were released on 14 April 2023, by MindMed. However, as of December of 2024, no phase III study of LSD in MDD was registered in Clinicaltials.gov.

In a phase IIa study by MindBio (ACTRN12623000486628), an open-label microdosing trial of LSD (MB22001), ranging from 5 μg to 15 μg per dosing day, was conducted in 20 patients with MDD and MADRS of 18–35 points at the time of screening [99]. The dose adjustment was based on patients’ experience and the severity of side effect(s). The initial dose of LSD was 8 µg for all patients. For patients who tolerated the initial LSD 8 µg, the LSD dose was increased by 1 µg per dosing day to a maximum dose of 15 µg per dosing day. For those who could not tolerate the initial 8 µg, the LSD dose was decreased to 5 µg (new baseline) on the second dosing day and then increased by 1 µg per dosing day from the third dosing day. The decision to adjust the dose was based on the participant’s self-report. The frequency of microdosing was twice a week for 8 weeks with at least one day apart in a 7-day period. The dosing was completed at home by patients. Patients with MDD who received MB22001for 8 weeks experienced a 60% reduction in depressive symptoms and 53% of patients achieved complete remission, with a mean reduction of 14.1 points on MADRS total score. The results of the study were posted on the MindBio website (https://www.mindbiotherapeutics.com, accessed on 20 December 2024).

Based on these phase IIa results, several phase IIb studies in MDD (ACTRN12624000128594), cancer patients with existential distress/depression (ACTRN12623000478617) [103], pre-menstrual syndrome, and pre-menstrual syndrome/pre-menstrual dysphoric disorder are underway. The protocol of the ACTRN12624000128594 trials has been published, with a dosing range from 4 to 20 µg per dosing day [104]. MB22001 may become the first psychedelic treatment approved for at-home use for MDD and other depressive disorders if phase III trials prove its efficacy and safety in the studied population.

##### 5-Methoxy-N, N-dimethyltryptamine (5-MeO-DMT)

5-MeO-DMT is a naturally occurring tryptamine that has been synthesized for medical research. It is a nonselective 5-HT receptor agonist with additional binding to serotonin, norepinephrine, and dopamine transporters. 5-MeO-DMT has higher binding affinity for the 5-HT_1A_ receptor than for the 5-HT_2A_ receptor [105], which is different from psilocybin and LSD, which both have higher affinity for the 5-HT_2A_ receptor than for the 5-HT_1A_ receptor [88]. Since oral 5-MeO-DMT is inactive, it is usually administered through smoking, vaporizing, intranasal, sublingual, intramuscular, intravenous, or rectal approaches. Smoking and vaporization are the most used methods. The effects after using 5-MeO-DMT are similar as those of other tryptamine psychedelics such as psilocybin and LSD. However, both 5-MeO-DMT and DMT are ultra-fast and short-acting psychedelics [106].

A cross-sectional study in a group setting with most patients with a diagnosis of depression or anxiety showed that using 5-MeO-DMT improved depression and anxiety symptoms. The improvement in depression or anxiety symptom severity was associated with the intensity of mystical experiences and the spiritual significance and personal meaning of the 5-MeO-DMT experience, but there were no associations between the symptom improvement and the intensity of physical/psychological effects during the 5-MeO-DMT experience [107].

Phase I and II studies were conducted by GH Research PLC (Dublin, Ireland), although some other companies were also interested in the development of this compound [105]. In a phase I/IIa study (NCT04698603) [108], the efficacy and safety of 5-MeO-DMT in a vaporized formulation (GH001) were assessed in eight adult patients with TRD. In phase I, four patients were assigned to a GH001 dose of 12 mg or 18 mg group. On day 7, remission rates (MADRS ≤ 10) were 50% for the 12 mg group and 25% for the 18 mg group, respectively. The mean change in MADRS total score from baseline to day 7 was −21.0 points for the 12 mg group and −12.5 points for the 18 mg group.

During phase II, all eight patients started with 6 mg, but could receive 6 mg, 12 mg, or 18 mg on a single dosing day. The next dose was determined by a peak experience and tolerability at a previous dose and given three hours apart. If a peak experience was not achieved and the patient did not have safety and tolerability concerns, the next dose of the drug would be increased after both the patient and the study clinician agreed. During phase II, 87.5% of patients achieved remission, which occurred from day 1, and the mean change in MADRS from baseline to day 7 was −24.4 points. With this positive study, GH Research moved forward with a phase II study of GH001 in PPD (NCT05804708) [109], and a phase IIb study in TRD (NCT05800860). The results of the phase IIb study became available in February 2025 [110]. The study found that GH001 had rapid onset of action, and at day 8 yielded a 15.2-point difference on the MADRS total score over placebo. The remission rate was 57.7% in patients receiving GH001 versus 0% in those receiving placebo.

##### N, N-Dimethyltryptamine (DMT)

Like psilocybin, LSD, and 5-MeO-DMT, DMT is a classic psychedelic and its hallucinogenic effects are believed to occur mainly through 5-HT_2A_ agonism, although DMT also binds to 5-HT_1A_, 5-HT_2C_, and 5-HT_1B_ receptors, serotonin and norepinephrine transporters, and other neurotransmitters [88,111]. DMT is an active ingredient in ayahuasca, with a short hallucinogenic duration ranging from 20 min to an hour. This shorter duration compared to psilocybin and LSD has driven researchers to study the role of DMT in psychiatric disorders as it may shorten the observation period during dosing days. For psilocybin and LSD, patients need to be under close observation and assisted therapy for about 8 h during dosing days.

A phase II open-label, single-ascending, fixed-order study was conducted by Universiade Federal do Rio Grande do Norte. Brazil (NCT06094907) to assess the feasibility and efficacy of inhaled DMT in patients with partial response in depression. Up to two inhaled doses (15 mg, followed by 60 mg) of DMT were administered on a single day one hour apart. The study was completed on 30 March 2024 with an enrollment of 14 patients. In a non-peer-reviewed article [112], patients with TRD and at least moderate symptoms (MADRS ≥ 20) had significant reductions in MADRS total score from day 1 to the end of one month (M1). The reduction in the mean MADRS score from baseline to day 7 was −22 points and −17 points at M1. At day 7, 83.33% of patients achieved response and 66.67% achieved remission. At M1, 66.67% and 50% of patients maintained response and remission status, respectively.

A phase I study was conducted by Yale University of the United States of America (NCT04711915) to assess the safety, tolerability, and efficacy of intravenous DMT in patients with TRD and healthy controls (HCs) in an open-label, fixed-order, dose-escalation design (0.1 mg/kg followed by 0.3 mg/kg) [113]. Both patients and HCs tolerated the medications well. HAMD-17 scores decreased significantly from baseline in participants with MDD on the next day after receiving 0.3 mg/kg DMT, with a mean difference of −4.5 points. DMT increased blood pressure, heart rate, anxiety, psychedelic effects, and psychotomimetic effects. However, these side effects resolved within 20–30 min after injection.

Small Pharma Ltd (London, United Kingdom). conducted a phase I/IIa study of DMT (SPL026) in patients with MDD and HCs (NCT04673383, ISRCTN63465876). In phase I (Part A), a randomized, double-blind, placebo-controlled, parallel-group, single dose-escalation trial, psychedelic-naive participants were randomized to placebo (*n* = 8) or four different escalating doses [9, 12, 17, and 21.5 mg intravenously (IV)] of SPL026 (*n* = 6 for each dose) in 5 min with psychological support from two therapists. All four SPL026 doses were safe and well tolerated without any serious adverse events. Results of the SPL026 phase I in healthy controls have been published [114].

In Part B, patients with a current major depressive episode (MDE) with moderate to severe symptoms and without taking an antidepressant received up to two single doses of SPL026, two weeks apart. The first dose was randomized, double-blind, and placebo-controlled, in which patients received either SPL026 21.5 mg or placebo. The second dose was open label to receive active SPL026 21.5 mg through I.V. injection in 10 min. During the randomized, double-blind, placebo-controlled phase, there were significant differences between active and placebo in reducing MADRS total scores at week 1 of −10.8 points, and at week 2 of −7.4 points. Of the 34 patients enrolled in the open-label phase, 57% of patients achieved remission at 3 months after a single dose of SPL026. For treatment response, 44% achieved response at week 1 and 35% achieved response at week 2. No apparent difference in antidepressant effect was observed between a one-dose and two-dose regimen of intravenous DMT. The intravenous dose of DMT for MDD was well tolerated by all patients who received an active dose [115].

Small Pharma Ltd. also conducted a phase Ib study to assess the safety and tolerability of single doses of SPL026 intravenous infusion in patients with mild to severe MDD with (*n* = 15) and without (*n* = 5) an SSRI (NCT05553691). A single dose of DMT 27.5 mg was given intravenously over 10 min. Decreases in depression scores from baseline were observed in both cohorts at Day 8, Day 15, and Day 29 following study drug administration. Overall, the DMT 27.5 mg was safe and well tolerated (ISRCTN10974027) [116].

##### 3,4-Methylenedioxymethamphetamine (MDMA)

MDMA is also known as the club drug “ecstasy”. Its psychoactive mechanism is believed to be through monoamine release, serotonin and norepinephrine reuptake inhibition, partial agonism of 5-HT_2A_, 5-HT_1A_, and 5-HT_2C_ receptors, and increase in oxytocin in blood levels [88]. It is the most studied psychedelic in psychiatric disorders without considering ketamine [117]. MDMA has been developed by the Multidisciplinary Association for Psychedelic Studies (MAPS) with a new name of Lykos Therapeutics (San Jose, CA, USA), receiving “breakthrough therapy” designation from the US FDA for MDMA-assisted psychotherapy for the treatment of Post-traumatic stress disorder (PTSD) in 2017 [118]. A phase III study demonstrated that MDMA-assisted psychotherapy was superior to placebo-assisted psychotherapy in the treatment of PTSD, with a large effect size [119]. Among patients with depressive symptoms, the MDMA-assisted psychotherapy was significantly superior to placebo-assisted psychotherapy in reducing depressive symptoms, with a moderate effect size. However, the US FDA declined to approve MDMA-assisted psychotherapy for PTSD in August 2024 due to ethical violations and data suppression. A search in Clinicaltrials.gov on 22 December 2024 with keywords of MDMA-assisted psychotherapy or MDMA with or without depression did not find any trial that is registered for MDMA in the treatment of depressive disorders. The majority of the registered trials were related to PTSD or anxiety disorders [120]. However, because of a moderate effect size for depressive symptoms in PTSD, MDMA-assisted psychotherapy or MDMA alone has the potential to be developed for depressive disorders.

#### 3.2.2. Glutamate-Related Agents

##### NMDA Receptor Antagonists

Esmethadone (REL-1017, d-methadone, and dextromethadone) is the opioid inactive (*S*)-enantiomer of racemic methadone. It is a noncompetitive NMDA receptor antagonist but has a weaker blockage effect compared to other NMDA receptor blockers such as ketamine, memantine, and MK-801 [121]. Compared to dextromethorphan, the antidepressant component in AXS-05, the affinity of esmethadone for NMDA receptor subtypes is close to that of dextromethorphan in some subtypes, and weaker than that of dextromethorphan in others. In addition to its NMDA receptor antagonism, esmethadone also has binding affinity to calcium and sodium channels, 5-HT receptors, serotonin transporters, and other neurotransmitters (Table 2).

With the success of AXS-05 and esketamine in treating depressive disorders, esmethadone (Relmada Therapeutics, Inc., Coral Gables, FL, USA) has been developed for the treatment of TRD with phase II and III trials [121,122,123]. In a phase IIa study [122], the onset of a significant antidepressant effect of esmethadone 25 mg/day and 50 mg/day versus placebo adjunctive therapy to a standard antidepressant occurred on day 4 in patients who had a major depressive episode (MDE) lasting from 8 weeks to 36 months and who failed 1–3 antidepressants for both adequate doses and durations. The effect size of esmethadone 25 mg/day or 50 mg versus placebo was large (≥0.8) on day 4 and day 14. However, in the first completed phase III study (NCT04688164) [123], esmethadone 25 mg/day as an adjunctive to a standard antidepressant was not superior to placebo adjunctive therapy to a standard antidepressant therapy in patients who had the same clinical presentation as those in the Phase III study [123]. In another completed phase III trial of esmethadone monotherapy versus placebo in MDD (NCT05081167), esmethadone 25 mg/day did not appear superior to placebo in reducing depressive symptoms after 4 weeks, with an MADRS score of −14.8 for esmethadone and −13.9 for placebo (clinicaltrials.gov). The difference in response and remission rate was also minimal. There are still two ongoing phase III efficacy trials investigating esmethadone as an adjunctive therapy in TRD (NCT06735079, NCT066011577). Clearly, the role of esmethadone as a monotherapy or adjunctive therapy in the treatment of MDD or TRD remains uncertain.

##### AMPA Receptor Modulator

The involvement of α-amino-3-hydroxy-5-methyl-4-isoxazolepropionic acid (AMPA) receptors in the antidepressant effect of ketamine was demonstrated in animal studies [124,125]. A preclinical study also showed that the activation of AMPA receptors by physiologically released glutamate enhances synaptic and cognitive functions [126].

**Osavampator**: Osavampator (TAK-653 and NBI-1065845) is a selective positive allosteric modulator of the AMPA receptors. In an animal study, TAK-653 showed an antidepressant-like effect [127]. In humans, Osavampator has been developed in the treatment of patients with MDD who have failed at least one antidepressant. A phase II study of Osavampator in TRD (NCT03312894) sponsored by Millennium Pharmaceuticals, Inc. (Takeda) was registered in Clininicaltrials.gov in 2018, but no patient was enrolled. In 2020, Takeda and Neurocrine Biosciences formed a collaboration to develop Osavampator for the treatment of TRD.

A phase II study of NBI-1065845 in TRD (NCT05203341) sponsored by Neurocrine Biosciences found that one dose of NBI-1065845 was superior to placebo in reducing depressive symptoms, with a −4.3-point mean difference in MADRS total score at day 28, and a −7.5-point difference in MADRS score at day 56 [128]. The positive results propelled the company to initiate a phase III study to assess the efficacy and safety of NBI-1065845 as an adjunctive treatment in patients with MDD (NCT06786624) [129].

#### 3.2.3. Opioid Receptor Antagonists

##### Aticaprant

For the efficacy and safety of aticaprant (JNJ-67953964) (Johnson and Johnson, New Brunswick, NJ, USA), a selective opioid κ receptor antagonist, 10 mg/day in MDD were first explored in patients with anhedonia and a history of depression (bipolar or unipolar depression) or anxiety disorders [130]. The study demonstrated that 8 weeks of aticaprant treatment significantly reduced the severity of anhedonia compared to placebo, with a significant increase in the activation of the ventral striatum during a reward anticipation task. However, there were no significant differences between aticaprant and placebo in reducing depression and anxiety symptom severities. A more recent phase II study of aticaprant in patients who had a diagnosis of MDD with an MDE lasting less than 12 months and who had failed an SSRI or SNRI at an adequate dose for at least 6 weeks found that 6 weeks of aticaprant was superior to placebo in reducing depressive symptoms measured by MADRS, with an effect size of 0.36 [131]. There are four phase III studies (NCT 05455684, NCT 05550532, NCT 065147742, and NCT 0663535) of aticaprant as an adjunctive therapy versus placebo in patients with MDD with moderate-to-severe anhedonia who have had an inadequate response to current antidepressant therapy with an SSRI or SNRI. The trial NCT 05455684 was completed on 18 September 2024, and the rest of the trials are ongoing.

##### Navacaprant

Navacaprant (NRMA-140) (Neumora Therapeutics, Inc., Watertown, MA, USA) is a potent, selective κ opioid receptor antagonist. Its selectivity for the κ receptor is 300-fold over that for the µ receptors. In contrast, the selectivity of aticaprant for the κ receptor is only 30-fold over that for the µ receptors [132]. The antidepressant effect of navacaprant is hypothesized to occur through blocking κ opioid receptors and modulating glutamatergic, GABAergic, serotonergic, and dopaminergic systems [133].

Navacaprant is developed by Neuroma Therapeutics Inc., Watertown, MA, USA and has been studied in patients with MDD in a phase II study and phase III studies. In the phase II study [133], patients with MDD and an HAMD-17 score of 14–30 points, and anhedonia with Snaith–Hamilton Pleasure Scale (SHAPS) ≥ 26 points were randomized to receive either navacaprant or placebo. In the total efficacy population, navacaprant significantly reduced HAMD-17 scores compared to placebo at week 4, but not at week 8. In the efficacy population with baseline HAMD-17 ≥ 22 points, navacaprant significantly reduced HAMD-17 scores compared to placebo at week 4 and week 8.

Phase III studies of navacaprant in MDD include KOASTAL-1 (NCT06029426), KOASTAL-2 (NCT06058013), KOASTAL-3 (NCT06058039), and a 52-week open-label extension study KOASTAL-LT (NCT06029439). The results of the KOASTAL-1 study were recently released, but navacaprant did not show superiority over placebo in primary and key secondary outcome measures [134]. Kappa opioid receptor antagonists may become an option for MDD, especially for those with severe anhedonia, if phase III studies confirm their efficacy and safety. However, their onset of action appears similar to monoamine-based antidepressants.

#### 3.2.4. Orexin Receptor Antagonist

Sleep disturbance is a diagnostic criterion of MDD and highly prevalent among patients with MDD. A bidirectional relationship likely exists between insomnia and depressive disorders [135]. Pre-clinical and clinical studies have shown that the orexin system is not only relevant to the regulation of sleep–wake cycles but also involved in reward processing and cognitive performance. Symptoms of anhedonia, sleep disturbance, and cognitive impairment in an MDE have propelled researchers to investigate the role of the orexin system in the pathogenesis of MDD and orexin receptor antagonists in the treatment of MDD [136].

##### Seltorexant

Seltorexant, also known as MIN-202/JNJ-42847922, developed by Janssen Research & Development, LLC and Minerva Neurosciences, is one of the several orexin-2 receptor antagonists approved for insomnia [137]. Its efficacy and safety in the treatment of TRD or NTRMDD have been assessed with randomized, double-blind, placebo-controlled trials. A single dose of seltorexant 10, 20, and 40 mg/day versus placebo was explored with a double-blind, four-way, crossover design in patients with MDD and persistent insomnia [138]. It was found that all doses were superior to placebo in improving sleep in a dose-dependent manner and 40 mg showed a trend toward significance in improving depressive symptoms.

In a phase Ib study conducted in the Netherlands, Belgium, and Germany in patients with MDD with or without ongoing antidepressant treatment [139], seltorexant, but not diphenhydramine, was significantly superior to placebo in improving core depressive symptoms. Another phase Ib study (NCT03374475) showed that 20 mg seltorexant monotherapy, but not 40 mg monotherapy, was significantly superior to placebo in reducing depressive symptoms [140]. The treatment benefit in the 20 mg arm remained significant even after sleep-related items were removed from HDRS scores. A phase IIb study (NCT03227224) also showed the superiority of seltorexant 20 mg as adjunctive therapy to an antidepressant over placebo in MDD at week 3, but not at week 6 [141].

A pivotal phase III trial (NCT04533529) was conducted to evaluate the efficacy and safety of seltorexant as an adjunctive treatment in adult and elderly patients who were moderately-to-severely depressed with significant sleep disturbance even with ongoing treatment with SSRI/SNRI treatment. The results indicated that seltorexant 20 mg was both a statistically significant and clinically meaningful improvement in depressive symptoms, based on the MADRS total score, and also improved sleep disturbance outcomes at day 43 [142]. However, the significant difference emerged only at the end of study week 7. Seltorexant was also safe and well tolerated in the study, with similar rates of common adverse events observed in both trial arms, consistent with previous seltorexant clinical trials. However, a similar phase III study in adults (NCT04532749) was terminated after enrolling 212 patients.

A multicenter, double-blind, randomized, placebo-controlled study is ongoing to evaluate efficacy and safety and the maintenance effect of seltorexant 20 mg as an adjunctive therapy to antidepressants in adult and elderly patients with MDD and insomnia symptoms (NCT06559306). Meanwhile, one phase III study (NCT04513912) and one phase II study (NCT03321526) comparing the efficacy and safety of seltorexant to quetiapine XR as an adjunctive therapy in TRD were completed. The results from the phase II study suggested that seltorexant was as effective as quetiapine-XR as an adjunctive therapy in the treatment of patients with TRD.

##### Filorexant and Suvorexant

In contrast to seltorexant, other orexin-2 receptor antagonists in MDD are less studied. A phase II study of filorexant (MK-6096) (Merck Sharp & Dohme LLC, Rahway, NJ, USA) 10 mg as an adjunctive therapy in patients with MDD and partial response to antidepressant monotherapy found that filorexant was not superior to placebo adjunctive therapy in reducing depressive symptoms (NCT01554176). A phase IV study of suvorexant (Institute for Advanced Medical Research, Alpharetta, GA, USA) 10–20 mg treatment for TRD (NCT02669030) was registered as a six-week, randomized, double-blind placebo-controlled trial investigating suvorexant as an augmentation therapy to antidepressant treatment for patients with MDD and residual insomnia. However, the status of the study is unclear.

The role of other orexinergic antagonists in depressive disorders is unclear. The future of this group of medications as a monotherapy for TRD or NTRMDD remains uncertain, although a phase Ib study demonstrated the superiority of seltorexant over placebo [140]. The onset of action of seltorexant in TRD appears like that of most other adjunctive therapies. However, its effect on insomnia may be particularly beneficial for patients with MDD and insomnia, especially for those experiencing early insomnia.

#### 3.2.5. Anti-Inflammatory Drugs

In addition to repurposing well-known anti-inflammatory drugs in depressive disorders, new drugs with anti-inflammatory properties are also being explored in the treatment of depressive disorders. A phase II study of JNJ-55308942, sponsored by Janssen Pharmaceutica N.V., Belgium, as a monotherapy in bipolar depression have been completed (NCT05328297). The purpose of the study was to evaluate the efficacy and safety of JNJ-55308942 versus placebo in bipolar depression. JNJ-55308942 is a potent, high-affinity antagonist of P2X7 receptors and attenuates P2X7 receptor-mediated interleukin-1β (IL-1β) release, which is believed to cause neuroinflammation in the central nervous system [143]. JNJ-55308942 has shown beneficial effects on animal models of depression related to stress and inflammation [144].

Another new anti-inflammatory drug from Janssen Pharmaceutical (JNJ-54175446) was studied in a phase I study in patients with MDD with or without ongoing antidepressant treatment (NCT02902601). The study was completed in 2017. A phase II study sponsored by Cambridge Clinical Trials Unit–Core (CCTU-Core), UK focusing on patients treated with one monoaminergic antidepressant drug (e.g., SSRI, SNRI, TCA) at an adequate dose for at least 6 weeks was registered (NCT04116606) in 2019, but the status of the trial is unknown. The mechanism of JNJ-54175446 is similar to that of JNJ-55308942 [143].

#### 3.2.6. Biomarker-Based ANTIDEPRESSANT Therapy

##### ALTO-100

Biomarker-based treatment is the goal of precision psychiatry. ALTO-100 was developed by ALTO Neuroscience (Mountain View, CA, USA) to treat MDD using brain biomarkers to identify patients who are most likely to respond to a specific drug. ALTO-100 is a small molecule and believed to work by increasing BDNF signaling and neuroplasticity in the hippocampus. To identify patients who are likely to respond to ALTO-100, the company used its AI-enabled biomarker platform to evaluate brain function biomarkers by analyzing EEG activity, neurocognitive assessments, wearable data, and other factors.

In a phase IIa open-label clinical trial (NCT05117632) [145], ALTO-100 showed favorable safety and tolerability in patients who had MDD with or without ongoing antidepressant treatment. Among the patients with a biomarker-defined cognitive profile, 61% of patients achieved a clinical response compared to 33% of patients without the biomarker. After six weeks of treatment, the biomarker-defined patient group achieved a 15.5-point mean reduction in MADRS scores compared to a 10.6-point reduction in the biomarker-negative cohort.

However, a large phase IIb randomized, double-blind, placebo-controlled, multicenter study (NCT05712187) [146] of ALTO-100 in adults with MDD did not find a significant difference between ALTO-100 and placebo in reducing depressive symptoms after 6-week treatment (altoneuroscience.com). The future of ALTO-100 is unclear. ALTO Neuroscience has other compounds including ALTO-203 (NCT06391593) [147] and ALTO-300 in development for treating MDD [148]. ALTO-300 has been evaluated with three phase II studies (NCT05118750, NCT05157945, NCT05922878). Two of them (NCT05118750, NCT05157945) have been completed. The results from these phase II studies will provide insight into whether ALTO-300 can be developed for depressive disorders.

## 4. Discussion

### 4.1. Application of Currently Available Antidepressants

The effectiveness study from STAR*D and efficacy studies from randomized, placebo-controlled trials provided evidence of the benefit of monoamine-based antidepressants in the treatment of MDD and TRD [5,6,7,8,9,10]. However, their limitations were reflected by low remission rates (30–40%) and slow onset of action (4–6 weeks or longer) [9,149]. There are many potential factors that may limit their effectiveness in the treatment of MDD [149,150]. In contrast, NMDA receptor antagonists including AXS-05, intranasal esketamine, ketamine infusion, and neurosteroids showed a faster onset of action although the effect size of AXS-05 in NTRMDD was similar to that of monoamine-based antidepressants.

Second generation antidepressants (SSRIs, SNRIs, and bupropion) are the first-line antidepressants for a current depressive episode of MDD recommended by the American Psychological Association (APA) [151], Veterans Administration/Department of Defense (VA/DOD) [152], and the Canadian Network for Mood and Anxiety Treatments (CANMAT) [153]. The VA/DOD guideline also recommends mirtazapine, trazodone, vilazodone, and vortioxetine as an initial therapy for a current MDE. Although citalopram was chosen as the first medication for MDD in the STAR*D for research purposes, based on the network meta-analysis [10], any SSRIs, SNRIs, or bupropion can be used as the first medication for a current MDE. There is no specific recommendation for a specific drug for a specific patient. However, previous treatment history, family history (genetic), psychiatric and medical comorbidities, and depressive subtypes such as melancholic versus anxious types should be considered when selecting the first medication for a current episode [150].

Atypical antidepressant (mirtazapine) and tricyclics (amitriptyline and clomipramine) were superior to placebo in reducing depressive symptoms in MDD [10]. Among the 21 antidepressants reviewed, amitriptyline and mirtazapine were the two most efficacious antidepressants, with acceptability comparable to placebo. Although both mirtazapine and nortriptyline monotherapy had low remission rates (20% and 12%, respectively) in stage II TRD patients in the STAR*D [6], their efficacy, which is comparable if not superior to SSRIs and SNRIs in non-TRD patients, suggests that both these medication monotherapies can be used as the first medication for a current episode of some patients with MDD. However, it is unclear what kind of patients are the best candidates for mirtazapine or tricyclics. For patients with severe comorbid anxiety symptoms and/or insomnia but without significant concern for weight gain, mirtazapine monotherapy may be a suitable option. Some evidence suggests that tricyclics are more effective than SSRIs in MDD patients with melancholic features. It is reasonable to consider mirtazapine or nortriptyline as an initial therapy for a current episode in patients who have a history of treatment failure with commonly used antidepressants due to side effects or lack of efficacy in previous episodes.

Only about 7% of patients who failed Level 3 treatment (mirtazapine, nortriptyline, lithium, or T3 augmentation therapy) in the STAR*D achieved remission with tranylcypromine [7]. Due to their potentially life threatening side effects such as hypertensive crisis and availability of ketamine/esketamine and repetitive transcranial magnetic stimulation (rTMS), MAOIs are unlikely to be used in the early stage of treatment for an MDE [5]. Similarly, the latest monoamine-based antidepressant, gepirone, is also unlikely to be used as a first-line medication due to its less robust efficacy and potential for QTc prolongation [24].

For medicinal and nutritional supplemental agents, St. John’s Wort was recommended as a first-line medication for mild depression, and a second-line medication for moderate depression, and Omega fatty-3 acid as a third-line medication for mild depression [153]. In contrast, SAMe was recommended as a third-line adjunctive therapy for mild to moderate depressive severity and L-methyl folate as a second-line adjunctive therapy for mild to moderate depressive episodes.

Agomelatine is the first non-monoamine antidepressant, but its utility in the treatment of MDD may be limited because of its small effect size in individual studies and in meta-analyses [26,27]. However, the network meta-analysis [10] showed efficacy in MDD similar to other commonly used first-line antidepressants and it has the best acceptability among 21 antidepressants. Meta-analyses of head-to-head comparison studies of agomelatine and other antidepressants also found that agomelatine was superior to other antidepressants, though with clinically insignificant differences based on Cohen’s D effect size [26,28].

Agomelatine is further limited by its approval by only a few regulatory agencies including the European Medicines Agency, Australia, and a few other countries. Agomelatine did not receive approval from the US FDA for treating MDD due to its less robust efficacy and the potential risk for liver toxicity. However, a nested case-controlled study involving more than 3 million patients who received agomelatine (*n* = 74,440) or other commonly used antidepressants (fluoxetine, paroxetine, sertraline, escitalopram, citalopram, mirtazapine, venlafaxine, duloxetine, and amitriptyline) found that neither agomelatine nor other antidepressants had an increased risk of liver injury compared to citalopram [154]. With its high acceptability and comparable efficacy to other commonly used antidepressants, agomelatine has been recommended as a first-line medication for MDD in the CANMAT 2023 updated guidelines for managing MDD [153].

In contrast, NMDA receptor antagonists may have a larger impact on the treatment of NTRMDD and TRD either as monotherapy or adjunctive therapy. The impact is mainly from the faster onset of action of AXS-05 compared to commonly used antidepressants and ketamine/esketamine compared to other adjunctive therapies. The efficacy of AXS-05 occurred in 1–2 weeks, which is much shorter than the 4–6 weeks of commonly used antidepressants [9,149]. It remains unclear if AXS-05 can become the first medication for a current MDE. Currently, in the United States, AXS-05 needs pre-authorization from insurance companies for treating NTRMDD. Moreover, the CANMAT 2023 updated guideline recommended AXS-05 as a second-line antidepressant like tricyclics for MDD [153].

The efficacy and safety of ketamine infusion and intranasal esketamine in the treatment of TRD are well established [155]. The rapid onset of action was observed in both ketamine infusion and intranasal esketamine. A large effect size of ketamine infusion relative to midazolam was found in patients who had failed at least three antidepressants [58]. However, in patients who had failed at least two antidepressants from two different classes and two augmentation treatments [59], the remission rate was only 5% at 24 h after a single ketamine infusion, and only 23% after six ketamine infusions. Without data on the efficacy and safety of ketamine infusion as monotherapy in NTRMDD, its role in NTRMDD remains unclear.

Intranasal esketamine in the treatment of TRD and MDD with SI also showed a faster onset of action compared to commonly used antidepressants, but the overall effect sizes at different assessment time points were small. The largest effect size was observed at 2–4 h after the administration and the smallest was reported at the end of assessment (week 3–4) with an SMD of −0.25 [48]. A similar pattern was also found in sub-group analysis of TRD or MDD with SI. The risk ratio for remission and response of esketamine versus placebo was 1.37 and 1.27, respectively, further indicating a small effect size [47].

In evidence-based medicine, a minimal SMD of 0.20 is considered clinically relevant. However, in real-world patients, the effect size of esketamine in MDD with SI and TRD may be smaller. Due to the short duration of its antidepressant effect [49], it remains unclear what the impact of esketamine will be in the treatment of depressive disorders. It is also true that intranasal esketamine can have a larger effect size in patients with NTRMDD. The most recent approval of intranasal esketamine as monotherapy for TRD will make esketamine an option for patients who have TRD and cannot tolerate an antidepressant. It remains unclear if esketamine will be further developed as a monotherapy for NTRMDD.

The role of neurosteroids in depressive disorders is likely limited to PPD because the efficacy of zuranolone as a monotherapy in MDD was either small or statistically insignificant compared to placebo [156,157,158]. The effect size of zuranolone in PPD based on two phase III studies was in the higher end of a small effect size range with an SMD of 0.49 [156], and the mean difference in HAM-D-17 total score exceeded four points [157,158]. In contrast, there were no large randomized, placebo-controlled studies of other antidepressants in PPD [159]. However, traditional antidepressants are likely to remain the mainstream drugs for moderate to severe PPD [160] even although zuranolone is the only oral medication specially indicated for PDD. The use of zuranolone may also further be limited due to the cost and restrictions imposed after taking the medication.

In summary, monoamine-based antidepressants, agomelatine, and AXS-05 have similar efficacy although AXS-05 has a faster onset of action. Ketamine infusion and intranasal esketamine may have a larger effect size in patients with NTRMDD. However, their side effects are different from relatively benign reactions to life-threatening events (Table 3). Citalopram over 40 mg per day, gepirone, and TCA need baseline and periodical ECG monitoring. Unstable hypertension, aneurysmal vascular disease, or arteriovenous malformation are contraindications to ketamine and esketamine treatments.

In addition to side effects, clinicians should also take cost and accessibility into account when selecting an antidepressant monotherapy for NTRMDD. As shown in Table 4, the cost of antidepressants differs widely even among the generic formations, and brand names of antidepressants cost much more than generic antidepressants. For ketamine infusion and intranasal esketamine, in addition to the cost, patients need to go to specialized clinics or doctor’s offices to receive treatments, with restrictions of driving or operating machinery until the next day after a restful sleep after each treatment, which can limit patients who do not have financial resources or social support to access these treatments.

### 4.2. Future Perspectives

In the future, psychedelics are likely to be developed as a monotherapy and adjunctive therapy for the depressive disorders. The importance of psychedelics in psychiatric disorders including depressive disorders is highlighted by a special issue of the *American Journal of Psychiatry* in January 2025 [161]. Among psychedelics, psilocybin has the most extensive data supporting its potential efficacy in MDD. The phase II data suggest that psychedelics have rapid onset of action, large effect sizes, and greater durability compared to NMDA receptor antagonists and traditional antidepressants. If phase III studies prove their efficacy and safety in depressive disorders either as a monotherapy or adjunctive therapy, the field will gain additional treatment options for depressive disorders. However, because of the unique experience associated with psychedelics and limitations of the current study design and the interpretation and translation of the current existing evidence, the implementation of psychedelic-assisted psychotherapy may face challenges in routine clinical settings [162,163].

The success of AXS-05 and esketamine in treating depressive disorders continues to inspire researchers and pharmaceutical companies to develop glutamatergic drugs for the treatment of depressive disorders. One example is esmethadone. In a phase II study of esmethadone in TRD [123], the onset of action of esmethadone relative to placebo was faster than that of most commonly used antidepressants, and the effect size compared to placebo was also larger than that of commonly used antidepressants. However, the first phase III study of esmethadone did not find a significant difference between esmethadone and placebo in reducing depressive symptoms in TRD [123]. Its future in depressive disorders relies on the outcomes of other phase III studies. Another example is Osavampator, a glutamatergic AMPA receptor modulator. If phase III studies of Osavampator prove its efficacy and safety in the treatment of MDD, it will become the first in its class of new antidepressants acting through AMPA receptor modulation.

Although navacaprant, an opioid κ antagonist, did not show efficacy in reducing symptom severity of MDD in a phase III study [134]; this outcome may not be surprising. An earlier imaging study of aticaprant for the treatment of anhedonia found that aticaprant was superior to placebo in reducing anhedonia. The reduction in anhedonia was associated with an increase in activation of the striatum during a reward performance task [130]. However, aticaprant did not have a significant effect on overall depression severity. It is worth noting that in this study, patients with anhedonia included patients with MDD, bipolar disorder, or non-affective conditions. Clearly, the future of navacaprant depends on further phase III studies. The future of aticaprant also hinges on its phase III studies. Even if phase III studies of navacaprant or aticaprant show superiority over placebo in MDD, these drugs may not be a game changer unless they offer a faster onset of action and/or larger effect sizes compared to currently available antidepressants. Our previous study found that patients with “pure anhedonia” without depressive mood in MDD are relatively rare [164]. Any attempt to develop these drugs specially targeting anhedonia may only limit their clinical utility and market value.

The future of orexin receptor antagonists is promising, but not particularly exciting. The onset of action of seltorexant (6 weeks) appears like that of traditional antidepressants. The mean difference from baseline to the end of the study between seltorexant and placebo (–2.6 points on MADRS total score) suggests that the antidepressant effect size of seltorexant is likely to be small. Since orexin receptor antagonists are primarily hypnotics, patients with MDD and comorbid insomnia may have more benefit compared to those without insomnia.

Bidirectional relationships between depression and inflammation support the development of anti-inflammatory drugs for the treatment of depressive disorders. Accumulating evidence suggests that the efficacy of anti-inflammatory drugs in depression may be associated with inflammatory burden. Studies based on inflammatory markers may yield more meaningful results. Although repurposing anti-inflammatory medications in depressive disorder has generated positive results, currently, there is no standardized recommendation or guidance on their use in depression. In the CANMAT 2023 update of the Canadian guideline for MDD, no specific anti-inflammatory drug was recommended [153]. New anti-inflammatory drugs under development may become a treatment option for patients with MDD and high inflammatory burden, provided that positive studies support their efficacy and safety.

One of the goals of precision psychiatry is to use biomarkers to predict treatment outcomes. However, challenges may arise related to the accuracy and reliability of biomarkers in assessing depressive symptom severity. Since depression severity is still subjectively measured with standardized scales/questionnaires, the strength of correlations between biomarkers and depression severity might be critical for success. The reason for failure of ALTO-100 in the phase II study remains unknown. However, Alto Neuroscience continues to search biomarkers to better predict treatment responses in MDD. According to a recent press release, ALTO-300 (a 25 mg formulation of agomelatine) will be studied in MDD by using ECG biomarkers [148].

## 5. Conclusions

New antidepressants with different mechanisms and possible faster onset, larger effect sizes, and longer durability under investigation are promising. However, traditional monoamine-based antidepressant monotherapies (SSRIs, SNRIs, bupropion, TCAs, and MAOIs) likely continue to be the primary options for depressive disorders worldwide, despite their slow onset and limited efficacy. With their relatively benign side effects and ease of access and use, SSRIs, SRNIs, bupropion, and agomelatine should be the first-line medications for a current MDE. In terms of which medication should be used as the first medication for a current MDE, clinicians should consider factors such as previous treatment response, family history, psychiatric and medical comorbidities, and symptom presentations. Meanwhile, clinicians need to understand the similarities and differences in mechanisms, efficacy, and side effects of all currently available antidepressants to maximize their benefits and minimize their harm.

NMDA receptor antagonists like AXS-05 and intranasal esketamine will play a bigger role, particularly in TRD, after their generic formulations become available. For neurosteroid antidepressants like zuranolone, their use is likely limited to patients with PPD even after their generic formulations become available.

For the future, psilocybin, LSD, 5-MeO-DMT, DMT, and MDMA have demonstrated antidepressant effects with rapid onset, large effect sizes, and long durability. If their safety and efficacy are validated with phase III studies, the treatment of depressive disorders is likely to change dramatically. Esmethadone (NMDA receptor antagonist) and Osavampator (AMPA receptor modulators) have shown potential in TRD, but inconsistent findings of esmethadone make its role in depressive disorder uncertain. The current findings from selective κ-opioid receptor antagonists and orexin receptor antagonists suggest that their onset of action and effect sizes are like traditional antidepressants as monotherapy and atypical antipsychotics as adjunctive therapy. Therefore, their impact on the treatment of depressive disorders is likely limited even if phase III studies demonstrate their efficacy and safety in depressive disorders. For biomarker-based antidepressant therapy, a recently failed phase II trial has raised concerns over its viability.

## Figures and Tables

**Table 1:** Mechanism of currently available antidepressants for depressive disorders.

Name	Dose (mg/day)	Mechanism
**Selective serotonin reuptake inhibitor and its related medications**		
Citalopram	10–40	5-HT↑ via inhibition of SERT
Escitalopram	5–20	
Fluvoxamine	25–300	
Fluoxetine	10–80	5-HT↑ via inhibition of SERT, NE↑ via inhibition of NET
Paroxetine	20–50	
Sertraline	25–200	5-HT↑ via inhibition of SERT, DA↑ via inhibition of DAT
Vilazodone	10–40	5-HT↑ via inhibition of SERT, 5-HT_1A_ partial agonist, 5-HT_3_ antagonist (for vortioxetine)
Vortioxetine	5–20	
Gepirone	18.2–72.6	5-HT_1A_ partial agonist and 5-HT_2A_ antagonist
**Serotonin–norepinephrine reuptake inhibitors**		
Venlafaxine	37.5–225	5-HT↑ and NE↑ via SERT and NET; SERT:NET = 30:1 for venlafaxine, SERT:NET = 14:1 for desvenlafaxine, SERT:NET = 10:1 for duloxetine, SERT:NET = 1:2 for levominacipran
Desvenlafaxine	50–100	
Duloxetine	30–120	
Levominacipran	40–120	
**Dopamine reuptake inhibitor**		
Bupropion	150–450	DA↑ via inhibition of DAT, NE↑ via inhibition of NET, antagonist of nicotinic acetylcholinergic receptor (nAChR)
**Norepinephrine reuptake inhibitor**		
Reboxetine	8–12	NE↑ via inhibition of NET
**Serotonin, norepinephrine, dopamine reuptake inhibitor**		
Toludesvenlafaxine	80–160	5-HT↑ via inhibition of SERT, NE↑ via inhibition of NET, DA↑ via inhibition of DAT
**Atypical antidepressants**		
Mirtazapine	15–60	5-HT↑ and NE↑ via pre-synaptic inhibition of α_2_ receptor, 5-HT_1A_ transmission↑ via antagonism of 5-HT_2_ and 5-HT_3_, Hypnotic: H_1_ receptor antagonism
Nefazodone	200–600	5-HT↑ and NE↑ via weak SERT and NET, 5-HT_2_ antagonist, a weak α_1_ adrenergic receptor antagonist
Trazodone	300–600	Simultaneous inhibition of serotonin transporters, 5-HT_2A_, and 5-HT_2C_ receptors. Hypnotic: antagonism of 5-HT_2A_ receptor, H_1_ receptor, and α-adrenergic receptors
**Tricyclic antidepressants**		
Amitriptyline	150–300	5-HT↑ via SERT > 5-HT_2_ antagonist > NE↑ with NET
Clomipramine	150–300	
Imipramine	150–300	5-HT↑ via SERT > NE↑ with NET > 5-HT_2_ antagonist
Doxepin	150–300	5-HT_2_ antagonist > NE↑ with NET > 5-HT↑ via SERT
Amoxapine	150–300	
Trimipramine	50–300	5-HT_2_ antagonist > 5-HT↑ via SERT > NE↑ with NET
Desipramine	75–300	NE↑ with NET > 5-HT↑ via SERT > 5-HT_2_ antagonist NE↑ with NET > 5-HT↑ via SERT > 5-HT_2_ antagonist
Nortriptyline	50–150	
Protriptyline	15–60	
Maprotiline	100–225	NE↑ with NET > 5-HT_2_ antagonist > 5-HT↑ via SERT
**Monoamine oxidase inhibitors**		
Phenelzine	30–90	Selective for MAO-B (lower doses) non-selective (higher doses), not reversible
Tranylcypromine	10–60	
Isocarboxazid	10–60	
Selegiline oral	1.25–10	Selective for MAO-A, not reversible
Selegiline transdermal	6–16	
Moclobemide	300–600	Selective for MAO-A, reversible
**NMDA receptors antagonists**		
Ketamine intravenous infusion	0.5–1 mg/kg, 40–60 min	Blocking pre-synaptic GABAergic NMDA receptors to increase release of glutamate and subsequently increase synthesis of BDNF and produce neuroplasticity.
Intranasal esketamine	56–84	
Dextromethorphan+ bupropion	45/105	Dextromethorphan: NMDA receptor antagonism + sigma-1 and mu opioid receptor agonism + inhibition of NET, and nAChR antagonism. Bupropion: inhibition of DAT and nAChR.
**Neurosteroids**		
Brexanolone Intravenous infusion	30, 60, 90, 60, 30 mcg/kg/h in 60 h	Positive allosteric modulation of GABA_A_ receptors to restore neuronal excitability and mood regulation
Zuranolone	50–40	
**Melatonergic agents**		
Agomelatine	25–50	Melatonergic agonism for sleep and 5-HT_2_c antagonism to increase NE and DA.
**Medicinal and nutritional agents**		
SAMe	400–600	Involved in the one-carbon metabolic cycle
St. John’s Wort	500–1800	Reuptake inhibition of 5-HT, NE and DA
Omega-3 fatty acids	1000–2000	Anti-neuroinflammatory; anti-oxidative stress; modulation of HPA axis; anti-neurodegeneration; neuroplasticity and synaptic plasticity; and modulation of neurotransmitter systems

**Table 2:** Mechanisms of potential future antidepressants for depressive disorders.

Psychedelics	
Psilocybin	5-HT_2A_ receptor partial agonist and binding to 5-HT_2C_ > 5-HT_1A_ > 5-HT_1B_ receptors; 5-HT↑ via inhibition of SERT.
Lysergic Acid Diethylamide (LSD)	5-HT_2A_ receptor partial agonist and binding to 5-HT_1A_ > 5-HT_2C_ > 5-HT_1B_ receptors; binding to D2 receptors; increase in glutamate release in frontal cortex; increase in functional connectivity and excitability in thalamus and cortexes.
5-methoxy-N, N-dimethyltryptamine (5-MeO-DMT)	5-HT receptor agonist with binding 5-HT_1A_ > 5-HT_2A_; 5-HT↑ via inhibition of SERT, NE↑ via inhibition of NET, DA↑ via inhibition of DAT.
N, N-Dimethyltryptamine (DMT)	5-HT receptor agonist with binding 5-HT_1A_ > 5-HT_2A_ > 5-HT_2C_ > 5-HT_1B_; 5-HT↑ via inhibition of SERT and increase in release; NE↑ via inhibition of NET; increase in other neurotransmitters.
3,4-Methylenedioxy-methamphetamine (MDMA)	5-HT↑ via inhibition of SERT; NE↑ via inhibition of NET; partial agonist of 5-HT_2A_, 5-HT_1A_, and 5-HT_2C_ receptors; increase in oxytocin in blood levels.
**NMDA receptor antagonist**	
Esmethadone (REL-1017).	Pre-synaptic GABAergic NMDA receptor blockade: release of glutamate↑; post-synaptic BDNF and neuroplasticity↑; binding to calcium and sodium channels, H_1_, M_5_, µ, 5-HT_2C_, 5-HT_5A_, 5-HT_7_ receptors, and NET.
**AMPA receptor modulator**	
Osavampator (TAK-653, NBI-1065845)	Selective positive allosteric modulator of the AMPA receptors.
**Opioid receptor antagonists**	
Aticaprant	Opioid κ receptor antagonism.
Navacaprant	Opioid κ receptor antagonism and modulating glutamatergic, GABAergic, serotonergic, and dopaminergic systems.
**Orexin receptor antagonist**	
Seltorexant, Filorexant Suvorexant	Orexin-2 receptor antagonists.
**Anti-inflammatory drugs**	
JNJ-55308942 JNJ-54175446	Antagonist of P2X7 receptors and attenuates P2X7 receptor-mediated IL-1β release

**Table 3:** Side effects of current antidepressants.

Antidepressants	Side Effects	
**Selective Serotonin Reuptake Inhibitors**		
Citalopram Escitalopram Fluoxetine Fluvoxamine Paroxetine Sertraline Vilazodone Vortioxetine	Sexual dysfunction Delayed ejaculation Anorgasmia Decreased libido Gastrointestinal Side effects Nausea and vomiting Diarrhea Constipation and Dry mouth (most common with paroxetine) Weight gain or loss of appetite (with fluoxetine, early stage of treatment) Central Nervous System Anxiety	Insomnia (fluoxetine) Sedation (paroxetine) Headache Nightmares and vivid dreams EPS symptoms (akathisia, …) Bleeding Hyponatremia Serotonin syndrome Discontinuation syndrome (especially with short half-life such as paroxetine and fluvoxamine) (fluoxetine is less likely to cause) Dizziness, nausea, weakness, insomnia, anxiety, irritability, headache.
Gepirone	QTc prolongation, minimal sexual side effects, does not cause weight gain	
**Serotonin–Norepinephrine Reuptake Inhibitors**		
Venlafaxine	Nausea, dizziness, insomnia, somnolence, dry mouth Sexual dysfunction Discontinuation syndrome Hypertension (Venlafaxine XR > 225 mg/day)	
Duloxetine	Nausea, dry mouth, constipation, fatigue, decreased appetite, sweating Sexual dysfunction Initial insomnia, irritability, anxiety, nervousness, and restlessness Mydriasis Hypertension	
**Dopamine Reuptake Inhibitor**		
Bupropion	Insomnia, headache, tremors, nausea, Increased irritability and agitation	Few sexual side effects Not cause increased appetite or weight gain.
**Atypical Antidepressants**		
Mirtazapine	Sedation Weight gain (increase appetite)	Neutropenia, thrombocytopenia, bone marrow supression (rare) Dizziness, dry mouth, constipation, disturbing dreams
Trazodone Nefazodone	Sedation Orthostatic hypotension Dizziness	Headache Priapism (rare)
**Tricyclic Antidepressants**		
Amitriptyline Clomipramine Doxepin Imipramine Trimipramine Desipramine Nortriptyline Protriptyline Amoxapine Maprotiline	Anticholinergic Dry mouth Constipation Urinary retention Blurred vision Confusion, Delirium Narrow-angle glaucoma Cardiac Effects Intraventricular conduction delay AV block	Flattened T waves Depressed ST segments Prolonged QT intervals Tachycardia Orthostatic hypotension Sedation Weight gain Sexual dysfunction Elevation of liver enzymes Discontinuation syndrome (mostly related to cholinergic and serotonergic rebound)
**Monoamine Oxidase Inhibitors**		
Phenelzine Tranylcypromine Isocarboxazid Selegiline Moclobemide	Orthostatic Hypotension, Dizziness, Reflex tachycardia Weight gain Sedation Hypertensive crisis (with tyramine rich food consumption) Headache, stiff neck, sweating, nausea, and vomiting Autonomic instability, elevated blood pressure, cardiac arrhythmia, coma, and death Sexual dysfunction Hepatotoxicity Pyridoxine deficiency Drug Interactions	
**Melatonergic Agents**		
Agomelatine	Headache, dizziness, somnolence, diarrhea, nausea, sedation, fatigue, and insomnia Hepatotoxicity Abdominal pain Lower risk of sexual dysfunction	
**N-Methyl-D-Aspartate antagonists**		
IV Ketamine IN Esketamine	Nausea, headache, blurred vision, drowsiness, dizziness, sedation, lethargy Psychiatric Dissociation Psychotomimetic Anxiety Perceptual disturbance Cognitive impairment	Cardiovascular/hemodynamic Hypertension Tachycardia Genitourinary tract symptoms Ulcerative cystitis Abuse potential Suicidal thought (in young adults)
Dextromethorphan + Bupropion	Dizziness, nausea, headache, diarrhea, somnolence, and dry mouth. Psychomimetic effects, weight gain, and increased sexual dysfunction or suicide-related adverse events were not reported.	
**Neurosteroids**		
Zuranolone Brexanolone	Somnolence, dizziness, headache, sedation, diarrhea, nausea, nasopharyngitis, urinary tract infection Presyncope (only for brexanolone)	
**Medicinal and nutritional agents**		
St. John’s Wort	Gastrointestinal irritations, allergic reactions, fatigue, restlessness	
SAMe	Gastrointestinal symptoms, sweating, vertigo, dizziness, tachycardia, restlessness, and anxiety	
Omega 3 fatty acids	Increased bleeding (increased risk combination with aspirin), headache, dizziness	

**Abbreviations**: CNS, Central Nervous System; EPS—Extrapyramidal Symptoms; IV, Intravenous; IN, Intranasal; QTc—Corrected QT Interval; SAMe—S-Adenosylmethionine.

**Table 4:** Formulation and weekly cost of antidepressants in North America.

Name	Dosage Form	Average Weekly Cost ($)
**Selective serotonin reuptake inhibitors and related agents**		
Citalopram (generic)	Tablet	0.93–1.86
Escitalopram (generic)	Tablet	2.18–2.32
Escitalopram (generic)	OD tablet	9.24–9.84
Fluoxetine (generic)	Capsule	2.32–2.98
Fluvoxamine (generic)	Tablet	2.65–7.94
Paroxetine (generic)	Tablet	2.28–4.69
Sertraline (generic)	Capsule	2.12–4.69
Vilazodone (Viibryd)	Tablet	21.88–29.12
Vortioxetine (Trintellix)	Tablet	20.64–22.41
**Serotonin Norepinephrine reuptake inhibitor**		
Desvenlafaxine (generic)	ER tablet	16.39
Duloxetine (generic)	DR capsule	6.84
Levomilnacipran (Fetzima)	ER capsule	26.88–30.60
Venlafaxine (generic)	ER capsule	1.28–2.63
**Dopamine reuptake inhibitor**		
Bupropion (generic)	SR capsule	1.08–1.61
Bupropion (generic)	ER capsule	1.02–2.05
**Atypical antidepressants**		
Mirtazapine (generic)	OD tablet/tablet	0.68–2.05
Trazodone (generic)	Tablet	1.02–2.03
**Tricyclic antidepressants**		
Amitriptyline (Aventyl)	Tablet	2.54–3.23
Clomipramine (generic)	Tablet	1.81–20.72
Desipramine (generic)	Tablet	6.65–19.96
Doxepin (generic)	Capsule	7.64–11.29
Impramine (generic)	Tablet	1.80–12.93
Nortriptyline (generic)	Capsule	10.91–14.54
Trimipramine (generic)	Tablet/capsule	10.98–20. 77
**Monoamine oxidase inhibitors**		
Moclobemide (generic)	Tablet	7.28 to 14.56
Phenelzine (Nardil)	Tablet	9.80–16.60
Tranylcypromine (Parnate)	Tablet	5.68–17.03
**Neuroteroid antidepressant**		
Zuranolone (Zurzuvae)	Capsule	7950
**N-methyl-D-aspartate receptor antagonists**		
Dextromethorphan/bupropion (Auvelity)	ER Tablet	144.20–179.43
Ketamine infusion *	Vial	5.20–15.59 + 800–2000 service fee for first 2–3 weeks
Esketamine (SPRAVATO) **	Intranasal Spray	W1: 1092–1365 + 4-h service fee W2–4: 1092–1638 + 4-h service fee W5–8: 546–819 + 2-h service fee W≥9: 273–819 + 2-h service fee

Note: * Performed in specialized clinic; ** Performed in specialized clinic and doctor’s office. Abbreviations: DR, delayed release; ER, extended release; OD, orally disintegrating; SR, sustained release. Reference: CADTH DRUG REIMBURSEMENT REVIEW Pharmacoeconomic Report. ESKETAMINE HYDROCHLORIDE (SPRAVATO) (Janssen Inc.) Indication: major depressive disorder in adults.

## Data Availability

No new data were created or analyzed in this study. Data sharing is not applicable to this article.

## Abbreviations

5-HT	5-hydroxytryptamine
5-MeO-DMT	5-methoxy-N, N-dimethyltryptamine
AEs	Adverse events
AMPA	α-amino-3-hydroxy-5-methyl-4-isoxazolepropionic acid
APA	American Psychological Association (2019)
AXS-05	Dextromethorphan and bupropion combination
BDNF	Brain-derived neurotrophic factor
CBT	Cognitive behavioral therapy
CGI-I	Clinical Global Impressions Scale
CANMAT	Canadian Network for Mood and Anxiety Treatments
DHA	Docosahexaenoic acid
DALYs	Disability-adjusted life years
DMT	N, N-Dimethyltryptamine
EPA	Eicosapentaenoic acid
ER	Extended release
GABA	Gamma-aminobutyric acid
GAD	Generalized anxiety disorder
HAM-A	Hamilton Anxiety Rating Scale
HAMD-17	Hamilton Depression Rating Scale—17 item
HPA	Hypothalamus-pituitary-adrenal
hs-CRP	High sensitivity C-reactive protein
IDS-C	Inventory of Depressive Symptomatology—Clinician
IL-1β	Interleukin-1β
LSD	Lysergic Acid Diethylamide
MADRS	Montgomery-Asberg Depression Rating Scale
MAOIs	Monoamine oxidase inhibitors
MAPS	Multidisciplinary Association for Psychedelic Studies
MDD	Major depressive disorder
MDE	Major depressive episode
MDMA	Methylenedioxymethamphetamine
NMDAR	N-methyl-D-aspartate receptor
NTRMDD	Non-treatment-resistant major depressive disorder
OR	Odds ratio
PPD	Postpartum depression
PHQ-9	Patient Health Questionnaire-9
PTSD	Post-traumatic stress disorder
rTMS	Repetitive transcranial magnetic stimulation
REMS	Risk Evaluation and Mitigation Strategy
SAT	Standard of care antidepressant therapy
SAMe	S-Adenosyl-L-Methionine
SGAs	Second-generation antidepressants
SHAPS	Snaith–Hamilton Pleasure Scale
SI	Suicidal ideation
SMD	Standardized mean difference
SNRIs	Serotonin–norepinephrine reuptake inhibitors
SSRIs	Selective serotonin reuptake inhibitors
STAR*D	Sequenced Treatment Alternatives to Relieve Depression
TCAs	Tricyclic antidepressants
T3	Triiodothyronine
TRD	Treatment-resistant depression
US FDA	United States Food and Drug Administration
VA/DOD	Veteran Administration/Department of Defense (2022)
YLDs	Years lived with disability
